# The relationship between self-reported preventive and curative orientations of dentists and oral healthcare services provided to Dutch young patients: An observational study

**DOI:** 10.1371/journal.pone.0306403

**Published:** 2024-07-05

**Authors:** Riet Hummel, Joost den Boer, Mariska Barendse, Geert van der Heijden, Wil van der Sanden, Josef Bruers

**Affiliations:** 1 Department of Oral Public Health (OPH), Academic Centre for Dentistry Amsterdam (ACTA), University of Amsterdam and Vrije Universiteit Amsterdam, Amsterdam, The Netherlands; 2 Zilveren Kruis, Zeist, The Netherlands; 3 KNMT, Royal Dutch Dental Association, Utrecht, The Netherlands; 4 Department of Dentistry - Quality and Safety of Oral Healthcare, Radboud University Medical Center, Radboud Institute for Health Sciences, Nijmegen, The Netherlands; Shahid Beheshti University of Medical Sciences School of Dentistry, ISLAMIC REPUBLIC OF IRAN

## Abstract

The aims of this study were to investigate the relationship between self-reported preventive and curative orientations of general dental practitioners (GDPs) and the oral healthcare services (OHS) they provided to patients under 18-years-old. And in addition, to determine which patient, GDP, and dental practice characteristics predicted the provision of preventive and curative care. GDPs in the Netherlands using dental software program Exquise (>2,000) were invited to participate in this study voluntarily. Participants completed a web-based questionnaire on characteristics of themselves, their dental practices, and on 20 hypothetical clinical situations concerning caries management. Based on their responses GDPS were classified for their preventive orientation, and their curative orientation. Data on the OHS provided to their young patients over the period 2013–2017 were automatically extracted from the patient files. Based on the annual frequency of provided care to regular patients over a period of 4 or 5 years, this was converted into 3 longitudinal care patterns regarding prevention and 3 longitudinal care patterns regarding curative care. Multinomial logistic regression analyses were conducted with a multilevel approach to correct for dental practices. The 37 participating GDPs provided data for 16,229 young patients. There was not a significant relationship between self-reported preventive orientations and preventive care patterns. The self-reported middle curative orientation was a predictor of the care pattern ‘curative treatment(s) in 1 year’ (OR 1.23 compared to nu curative treatments; 95% CI 1.02–1.48). The self-reported high curative orientation was a predictor of ‘curative treatments in several years’ (OR 1.90; 95% CI 1.27–2.85). Common characteristics predicting (p<0.05) both regular preventive care and curative treatments in several years were patient related: age 4–9 and 10–12, low-income neighborhood, 5 years included in study. GDP and dental practice related predictors were: the GDP could fulfill the care demand by working overtime, small dental practice (≤2,000 patients), and practice policy on the provision of care to young patients. This showed that the variation in provided care was partly supplier-driven instead of patient-centered.

## Introduction

In the Netherlands, the costs for oral healthcare services (OHS) are covered for all patients up to the age of 17 years from a compulsory standard healthcare insurance and the payment system is fee-per-item. Data on provided OHS showed variation between general dental practitioners (GDPs) in the preventive and curative care they provided to their young patients [1–3]. This may, of course, have been due to differences in the caries risk of patients [2]. However, there was uncertainty on the most effective preventive and curative management of dental caries in young patients [4], and GDPs may have differed in their opinions on good care. This may indicate that appropriate care was not always provided. Some patients may have received less care than necessary and others more [5]. Ultimately, this could have resulted in poorer oral health, unnecessary high(er) costs, or both [6]. Differences in the indication for preventive and curative care can be revealed by presenting GDPs hypothetical clinical situations and asking them what their management approach would be [7, 8].

Hummel et al. [3] have used data on provided OHS to describe longitudinal care patterns based on the number of years a patient received preventive or curative care. They have included 16,229 patients from 37 GDPs. The patients received regular OHS over a period of at least 4 to a maximum of 5 years. They have been divided into 3 preventive care patterns: no preventive care (9.2%), incidental preventive care (2 or more years without preventive procedures; 35.1%) and regular preventive care (not more than 1 year without preventive procedures; 55.7%). And subsequently in 3 curative care patterns: no curative treatments (41.6%), curative treatment(s) in 1 year (26.5%) and curative treatments in several years (31.9%).

The distribution of care patterns has shown considerable variation between dental practices. There were major differences in the preventive care provided but there was little variation within dental practices. That is, GDPs seemed to have applied the same preventive approach to most of their young patients, irrespective of the curative care provided. This indicated that the (frequency of the) provided preventive care was mainly determined by the provider and could not be explained by the health situation of their patients [3]. This has been known as supply-sensitive care [9]. The variation in curative care patterns could not be explained. To provide such an explanation, more information was required like the clinical decision-making of GDPs on when to intervene curatively in caries lesions.

Mercuri and Gafni reported that the reasons of treatment variation are difficult to identify. Patients’ characteristics are assumed to mainly drive variation in choice of treatment. Where available, this choice of treatment should be based on clinical guidelines [10]. Such provision of care is known as effective care [9]. However, preferences of GDPs and circumstances of dental practices can also play a role [11, 12]. GDPs may, for example, have different views on ’good care’ to prevent and treat dental caries in young patients, and have different styles of practice. Some of them are oriented towards prevention and provide more preventive care; others are oriented towards curative treatments and provide relatively more curative care [11]. It is well-known that there are major differences in the Netherlands in the management of caries in the primary dentition (monitoring, a preventive, a non-restorative, or a curative approach) [13, 14]. GDPs have been shown to apply different thresholds for restorative treatment, and so differ in the stage at which they reported they would intervene restoratively for caries in the permanent dentition [15]. In addition, Lewis et al. [16] and Rindal et al. [17] reported that the caries lesion depth at which GDPs actually intervened restoratively not always corresponded with the self-reported restorative treatment threshold. This raised the question to what extent the self-reported preventive and curative caries management approaches of GDPs are reflected in data on provided OHS. In this context, caries management equals the procedure(s) a GDP would practice in a certain clinical situation. The caries management approach can be preventively oriented or curatively orientated, or both, while at the same time the amount of both can vary.

The aim of this study was twofold. Firstly, to investigate the relationship between self-reported preventive and curative orientations of GDPs and the OHS provided in their dental practices to patients under 18 years of age; and secondly, to determine which patient, GDP and dental practice characteristics predicted the provision of preventive and curative care.

## Methods

This observational study was conducted in the Netherlands using cross-sectional data from a questionnaire (2020) and longitudinal data for provided OHS (2013–2017). The study was set up by the Academic Centre for Dentistry Amsterdam (ACTA) and was carried out in collaboration with the Royal Dutch Dental Association (KNMT), the Radboud University Medical Center, and software supplier Vertimart (supplier dental software program Exquise).

### Participants

GDPs were recruited to participate voluntary in this study. The study was announced in the Dutch dental magazine (Nederlands Tandartsenblad) in August 2020 and more detailed information was posted on www.staatvandemondzorg.nl, a website of KNMT [18]. Soon afterwards, software supplier Vertimart sent an e-mail to their customers (>2,000) to invite them to participate. This approach was adopted as Vertimart was not allowed to share customer data with researchers due to Dutch privacy law and regulations. Furthermore, the research has been brought to the attention of two large groups of dental practices and to members of the Dutch Association of Pediatric Dentistry (NVvK). Vertimart sent another invitation for participation in October 2020. Potential participants signed up via a register website from trusted data management and data protecting agency KBA (www.kbanijmegen.nl). Firstly, the GDPs had to answer some questions to confirm that they met the inclusion criteria. If they did, they received a confirmation mail and an information letter about the study. Informed consent was given in the questionnaire. KBA sent reminders to the registered GDPs in September and November 2020, and subsequently tried to phone all of them to ask whether they had already participated, whether they encountered problems, or what reasons they had not to participate anymore. The medical Ethics Review Committee of VU University Medical Center waived the requirement for informed consent for patients as they judged that the Medical Research Involving Human Subjects Act did not apply to this study (number 2019.551). The medical Ethics Review Committee of VU University Medical Center (Vrije Universiteit Amsterdam) is registered with the US Office for Human Research Protections (OHRP) as IRB00002991. The FWA number assigned to VU University Medical Center is FWA00017598.

Inclusion criteria for participation were that the dentist was working as GDP in the Netherlands, had been practicing in the same dental practice since 2013, had been using dental practice software program Exquise since 2013, and had performed routine oral examinations (ROEs) in at least 50 young patients (under 18 years of age) per year (2013 through 2017). However, participation was not possible if the GDP was working in a group of dental practices with multiple locations where the provided OHS had not been registered per location.

### Questionnaire

The questionnaire consisted of questions on clinical cases, professional practice of the GDP, the work and dental practice situation, and general characteristics of the GDP. The translated questionnaire is provided in S1 Questionnaire.

The questionnaire was modified from a formerly used questionnaire by Bruers [11]. He aimed to study variables related to differences in provided OHS by GDPs expressed as certain styles of practice, like preventive and curative. Clinical cases to collect information on opinions about the management of dental caries in the primary and permanent dentition were added. For the primary dentition, 4 different hypothetical clinical cases were shown considering cavities of increasing severity in a single molar. The descriptions and management options were reused from Tickle et al. [7] with minor adjustments to better match the Dutch situation. Photographs of the caries lesions were obtained from the department of pediatric dentistry from ACTA. The combination of the photographs and the descriptions of the cases were discussed with a pediatric dentist and considered realistic. For the permanent dentition, there were 2 cases considering caries in an occlusal surface and 2 cases in an approximal surface. Each case consisted of four successive stages in the caries progress (from discoloration / enamel caries up to caries in the middle third in the dentin) in either a patient with low caries risk or a patient with high caries risk. The descriptions of occlusal lesions were reused from Mejàre et al. [19]. The photographs of different stages of caries lesions in an occlusal surface were reused from Espelid et al. [20] and drawings of different stages of caries lesions in an approximal surface from Mejàre et al. [19]. In total, 20 clinical situations concerning dental caries were presented: 4 in the primary dentition, 4 in an occlusal surface in a low-risk patient, 4 in an occlusal surface in a high-risk patient, 4 in an approximal surface in a low-risk patient, and 4 in an approximal surface of a high-risk patient. GDPs were asked what their caries management approach would be. Each situation had a list of possible caries management options, which were possible procedures to choose from. Multiple answers were possible. The situations in the primary dentition offered 13 options: monitoring, prescribing/advising a painkiller, prescribing antibiotics, oral hygiene instruction, professional fluoride application, non-restorative cavity treatment (NRCT, this may include opening the cavity for better accessibility), ART-restoration, restoration, prefab crown/ Hall technique, opening the pulp chamber and allowing the tooth to drain, pulpotomy, tooth extraction, refer for tooth extraction. For the approximal surfaces in the permanent dentition 4 options were offered: monitoring, oral hygiene instruction, professional fluoride application, and restoration. The situations concerning occlusal surfaces had the same options with the additional option ‘sealing’. Estimated percentages of cavitated lesions were asked for 3 different stages of the caries process: enamel-dentin border reached, outer third of the dentin, middle third of the dentin. The estimated progression rate of caries lesions was asked for 4 situations: lesion progression in enamel from the outer layer to the enamel-dentin border, and lesion progression in dentin from the enamel-dentin border to the inner third part of the dentin; both in a low risk and high-risk patient. To determine the style of practice, descriptions were given of four types of dentists: restorative, preventive, directive, and communicative. GDPs were asked in which type they recognized themselves mostly. The descriptions were derived from de Vries et al. [21] and den Dekker [22] and in terms of formulation slightly adapted to the current situation in the provision of OHS.

Although the questions had already proven themselves in previous research, the questionnaire was still tested by 3 GDPs for further validation. Based on their feedback, minor adjustments were implemented, mainly in the order of the questions. Then, the questionnaire was processed into a web survey.

### Provided care

The data on the provided OHS was automatically extracted from the patient files. To this end, Vertimart had built an application into Exquise, their electronic patient record (EPD), resulting in the selection of all data on OHS provided in the period 2013 through 2017 to patients who met the inclusion criterion. This implied that the patients had to be born between 1 January 2000 and 31 December 2012. So, they were 0 to 17 years old during the study period and insured for the costs of OHS throughout this entire period.

The available information included procedure codes of all provided OHS, year of birth, gender, income characteristics of the neighborhood linked to the 4-digit postal code of the home address, and an identification code that precluded traceability of individual patients. Information added to each procedure code was the date of treatment, the tooth number (if applicable), and a non-traceable identification code for the dental practice that provided the procedure.

### Data collection

The data collection concerning the questionnaire and provided care started on 31 August 2020 and ended on 19 February 2021. This was done on behalf of the KNMT by the trusted data management and data protecting agency KBA in Nijmegen via the aforementioned application in the EPD of software supplier Vertimart. In this application, GDPs started answering the questionnaire. When this was completed, they sent their responses to KBA followed by sending the data of the provided OHS in their dental practices. Vertimart programmed the data collection in the Exquise software in such a way that KBA only received research data containing non-traceable identification codes for GDPs and patients. KBA linked both datasets to the same non-traceable identification code of the GDP and sent them to the researchers.

### Data processing—Questionnaire

The questionnaire responses were processed and categorized. The number of GDPs per category was taken into account for the classification into categories to enable the analyses.

*Orientation* indicates whether a GDP would apply a more passive or monitoring approach, a more active approach, or somewhere in between. This was determined both for the preventive and curative orientation.

The *preventive orientation* of a GDP was determined by summing all chosen preventive caries management options for the 20 clinical situations. As multiple answers were possible, the sum of preventive options could range from 0 up to 48. This could be either or both oral hygiene instruction and professional fluoride application, as well as sealing in situations concerning caries in the occlusal surface of a permanent tooth. The categorization of the preventive orientation was based on quartiles and was ‘low’ (lowest quartile), ‘middle’ (2 middle quartiles) or ‘high’ (highest quartile).

The *curative orientation* of a GDP was determined by counting the number of situations in which the GDP would intervene curatively (up to 20 clinical situations). This was the case if a restoration was chosen as caries management option for the situations in the permanent dentition, and in the primary dentition if one or more of the following options were chosen: NRCT, ART restoration, restoration, prefab crown/Hall technique, opening the pulp chamber and allowing tooth to drain, pulpotomy, extraction, and referral for extraction. Then, according to the distribution of the curative orientation a GDP was categorized as ‘low’ (lowest quartile), ‘middle’ (2 middle quartiles) or ‘high’ (highest quartile).

*Rate of lesion progression*: For the 4 situations where an estimate of the rate of lesion progression was asked, the rankings of the chosen answer options were considered as points and summed. When the time for progression from the first into the second stage was estimated to be 3–6 months, 5 points were given; 6–12 months 4 points; 12–24 months 3 points; 24–48 months 2 points; and >48 months 1 point. Subsequently, ‘the estimated rate of lesion progression’ was divided into 2 categories based on the average: ‘estimated rate higher than average’ and ‘estimated rate lower than average’.

*Percentage of cavitated lesions*: For 3 different stages in the caries process, an estimate of the percentage of cavitated lesions was asked. In these cases, the ranking numbers of the answers were also regarded as points and summed. When the percentage of cavitated lesions was estimated to be 0–25%, 1 point was given; 25–50% 2 points; 50–75% 3 points; 75–100% 4 points; and 100% 5 points. ‘The estimate of the percentage of cavitated lesions’ was divided into 2 categories based on the average: ‘estimated percentage lower than average’ and ‘estimated percentage higher than average’.

*Relationship between the actual depth of a caries lesion and the image on a bitewing radiograph*: The 3 answer options were reduced to 2 categories: the radiographic depth of a caries lesion underestimates the actual depth ‘yes’ or ‘no’.

*Experiences obstacles in the treatment of young children*: The questionnaire included 3 statements about the treatment of young children with a 5-point response scale (strongly disagree, largely disagree, neither disagree nor agree, largely agree, strongly agree). These 5-point scales were converted to obstacle yes/no. This was the case if a GDP strongly or largely disagreed with the statement ’Treatment of children derives satisfaction’, or if a GDP largely or strongly agreed with the statements ’Treatment of young children is difficult’ and/or ’The fees for the treatment of young children are inadequate’. The experienced obstacles were added and the variable was then converted to experiences an obstacle ‘yes’ or ‘no’.

*Use of guidelines*: The question ’Do you use one or more clinical guidelines in the treatment of young patients?’ had 4 answer options: never, sometimes, regularly, and often. These were reduced to 2 categories: ‘never/sometimes’ and ‘regularly/often’.

*Professional activities*: The numbers of hours per month that a GDP reported spending on post graduate courses, peer consultation and reading dental literature were added. The study of den Boer et al. [23] showed that the average time GDPs spent on professional activities was about 10 hours per month. The variable was divided into 2 categories based on this average.

*Diagnostic methods*: The number of diagnostic methods that a GDP could use for the diagnosis of caries lesions in young patients was asked. For each method, the answers were dichotomized into no/yes. The latter was a combination of the answer options sometimes, regularly, often, and always. All methods with yes were summed and two categories were formed based on the average.

*Style of practice*: GDPs had to indicate which style they most identified with: restorative, preventive, directive, or communicative. These styles were reduced to 2 categories: communicative style of practice ‘yes’ or ‘no’.

*Age first ROE*: The answer options ‘<1 year’ and ‘between 1 and 2 years’ were merged, as were the answer options ‘between 2 and 3 years’ and ‘between 3 and 4 years’.

*Minimum age for restorative treatment*: The answers ’no’ and ’no, it depends on the child’s cooperation’ were merged.

*Number of patients dental practice*. This refers to the number of patients that used to visit the dental practice one or more times per year. This variable was divided into 3 categories: ‘up to 2,000 patients’ (small practice), ‘2,001–4,000 patients’ (medium practice size), ‘more than 4,000 patients’ (large practice).

*Number of patients GDP per week in participating dental practice*: Based on the average number of patients, this was converted into 2 categories: ‘less than 100’ and ‘100 or more’.

*Percentage of young patients per dental practice / per GDP*: In the Netherlands, 20% or less is common [23]. The responses were divided into 2 categories: ‘20% or less’ and ‘more than 20%’.

*Personal workload*: The answers ‘I am able to fulfill the care demand within regular working hours’ and ‘I am not busy enough and could fulfill a larger care demand’ were combined. Two categories remained: ‘able to fulfill the care demand within normal working hours’ versus ‘able to fulfill the care demand by working overtime’.

*Task delegation*: For 8 procedures, GDPS were asked whether these were (also) executed by a prevention assistant and/or dental hygienist in their dental practice. The number of tasks that could be delegated were added and then divided into 3 categories: ‘0’, ‘1–4’, ‘5–8’.

*Practice policy on the provision of care to young patients*: The GDPs were asked whether agreements were made in their dental practices about the provision of care to children. The answers ’no’, ’I don’t know’ and ’not applicable’ were combined to ‘no’.

The *year of graduation*: The cut-off point for the division into 2 categories was about halfway through the range: ‘before 1995’, and ‘in 1995 or later’.

*Place of graduation*: Due to practical reasons concerning the distribution, the answers ‘Utrecht’ and ‘abroad’ were combined into the category ‘elsewhere’. The dental school in Utrecht was closed in 1988.

*The average number of inhabitants per dentist in a region* (dentist ratio) was determined by means of the 2-digit postal code of the dental practice. The KNMT provided a file in which the average dentist ratio was calculated for all 2-digit postal code areas in the Netherlands. The dentist ratio was divided into 3 categories based on the national average ratio (approximately 2,000 inhabitants per dentist) with a range of 200: ‘<1,800’, ‘1,800–2,200’, ‘>2,200’.

### Data processing—Provided OHS

The provided OHS were converted into longitudinal preventive and longitudinal curative care patterns as described in an earlier article by Hummel et al. [3].

*The preventive care pattern* was based on the number of years in which a patient received 1 or more of the following preventive procedures: oral hygiene instruction, professional tooth cleaning, professional fluoride application and/or sealant(s). Subsequently, patients were assigned to 1 of 3 preventive care patterns: no preventive care, occasional preventive care (2 or more years without preventive care), or regular preventive care (not more than 1 year without preventive care).

*The curative care pattern* was based on the number of years with 1 or more of the following curative procedures: NRCT, restoration in a primary tooth including confection crown, restoration in a permanent tooth, and extraction of a primary or permanent tooth. Patients were then assigned to 1 of 3 curative care patterns: no curative treatments, curative treatment(s) in 1 year, or curative treatments in several years.

*Mean number of procedures per patient per year*: This was calculated for the 4 preventive (oral hygiene instruction, professional tooth cleaning, professional fluoride application, session concerning 1 or more sealants) and the 3 curative procedures (NRCT, restoration, and tooth extraction).

*Age category patient*: The year of birth was converted to age on 1 January 2013. The patients were then divided into age categories approximating a primary, mixed, or permanent dentition in the first 2 years of the study period.

*The average income in the patient’s neighborhood* was determined by means of the 4-digit postal code of the home address of the patient. Statistics Netherlands has published a table with the average income per neighborhood [24]. Some 4-digit postal codes appeared in more than one neighborhood. In that case a weighted average was calculated. The categories for income categories were based on the average income: lowest 30%, middle 40%, and highest 30%. For some postal codes were no income data provided, these were set to ‘unknown’.

*Number of years included in study*: Only patients who had been a regular patient in the same participating dental practice for 4 to 5 years were included. Regular meant that the patient had visited this dental practice for a ROE in (almost) every consecutive calendar year during the study period. They were included 4 years in the analyses if they had visited the same dental practice for at least 1 ROE in 4 consecutive calendar years in the period 2013–2016 or 2014–2017, or if a maximum of 1 calendar year was missed within that 4-year period. Patients were included 5 years if they had at least 1 ROE in all 5 calendar years (2013–2017), or if a maximum of 1 calendar year was missed within that 5-year period.

### Data analyses

Separate analyses were conducted for prevention and curative care. It was shown graphically to what extent the provided preventive and curative care matched the self-reported preventive and curative orientations. The differences in the distribution of the care patterns (ordinal categories) between the groups with different preventive and curative orientations were tested with Kruskal Wallis tests. The differences in the mean number of procedures per patient per year between the groups were tested with one-way ANOVA and post hoc Tukey tests. These analyses were carried out in IBM SPSS Statistics (version 27).

To identify the predictors of the preventive and curative care patterns, multinominal logistic regression analyses were conducted with a multilevel correction for the nested structure in the data (that is patients nested in dental practices). The multilevel corrections adjusted the standard errors and the corresponding statistical tests. Ordinal variables were used for the preventive and curative orientations. Due to the small number of participating GDPs, the analyses were based on the patient data. The multinomial logistic regression analyses were carried out in Mplus (version 8) [25]. Although we built the model block-wise to ensure that the model was stable, we only report the final model with all explanatory variables, as we opted for exploratory analyses. The clusters used for the block-wise model and the variables within them are shown in Table 1.

**Table 1:** Clusters and variables multinomial logistic regression analyses with multilevel correction for dental practice.

Cluster	Variables in both preventive and curative model	Additional in preventive model	Additional in curative model
**Dependent variable**		• Longitudinal preventive care pattern	• Longitudinal curative care pattern
**Orientation of the GDP**		• Self-reported preventive orientation	• Self-reported curative orientation
**Background characteristics patient**	• Age category January 2013		
	• Gender patient		
	• Income category neighborhood		
	• Number of years included in study		
**Personal characteristics GDP**	• Gender GDP		
	• Year of graduation		
	• Place of graduation		
**Caries-related opinions GDP**			• Estimated lesion progression rate
			• Estimated percentage cavitated lesions
			• Estimated depth of radiographic lesions
**Professional beliefs and behaviors GDP**	• Style of practice	• Experiences an obstacle in the treatment of young children	• Age first ROE
	• Use of clinical guidelines		
	• Use of (additional) diagnostic methods	• Minimum age for a restorative intervention	
	• Registration of caries risk as part of a ROE		
**Professional characteristics GDP**	• Owner of the dental practice	• Number of patients per week GDP	
	• Percentage of patients <18 yrs old GDP		
	• Refers children for care or (specialized) treatment		
	• Personal workload		
	• Professional activities		
**Characteristics dental practice**	• Number of patients dental practice	• Percentage of patients <18 years old dental practice	
	• Practice policy on the provision of care to young patients	• Number of tasks executed by a dental hygienist and/or prevention assistant	
	• Mean number of inhabitants per GDP in region		

## Results

### Participants

Ninety-six GDPs registered for participation, 51 GDPs started on the questionnaire and 44 of them completed it. Thirty-seven GDPs (38.5% of the 96 registered GDPs, and about 2% of all potential participants) also delivered data on provided OHS, these were the included participants in this study. The reasons given by GDPs for not participating after registration were: no time, participation is too much work/too complicated, the dental practice is ceased, wrong dental software, the GDP passed away, and other personal circumstances.

The characteristics of the participating GDPs are described in the supporting information (S1 Table). Our participants were older compared to all GDPs in the Netherlands (mean age 54 versus 47), more often male (68% versus 55%), less frequently educated abroad (3% versus 19%), and more frequently owner of a dental practice (92% versus 60%) [26].

### Questionnaire

The descriptive results of the questionnaire are shown in the S1–S7 Tables in the supplementary information. S1 Table shows the general personal and professional characteristics of the participating GDPs. The opinions of GDPs on the management of dental caries in the primary dentition are shown in S2 Table, in an occlusal surface of a permanent tooth in S3 Table, and in an approximal surface of a permanent tooth in S4 Table. The mean number of chosen preventive options over the 20 hypothetical clinical situations was 31 with a range of 11 to 44. Cut-off points for the categorization of the preventive orientation were around the 25th and 75th percentiles. The category was ‘low’ in case of 11–25 chosen options, ‘middle’ in case of 26–35 options and ‘high’ in case of 36–44 options. This resulted in 10 GDPs being categorized as ‘low’ (27.8% of all patients), 15 as ‘middle’ (36.2%), and 12 as ‘high’ (36.0%). The average number of situations in which GDPs would intervene curatively was 12 with a range of 8 to 17. Again, cut-off points for the categorization were around the 25th and 75th percentiles. The curative orientation was ‘low’ if a GDP would intervene curatively in 8–11 situations, ‘middle’ in 12–13 situations or ‘high’ in 14–17 situations. The distribution of these categories was: ‘low’ 17 GDPs (43.4% of all patients), ‘middle’ 12 GDPs (37.2%), and ‘high’ 8 GDPs (19.4%). The estimated severity of caries lesions are described in S5 Table. The totals for the estimated rate of lesion progression from the first to the second stage in different situations ranged from 10 to 16 with an average of 13.2. The totals for the estimated percentages of cavitated lesions in different stages of the caries process ranged from 4 to 12 with an average of 7.5. Professional opinions and behaviors of the GDPs are shown in S6 Table. The total number of diagnostic methods a GDP could use for the diagnosis of caries lesions ranged from 3 to 7 and the average was 6.2. Eventually the characteristics of the participating dental practices are described in S7 Table.

Some variables have been excluded from the analyses. The Dutch quality register of dentists (KRT) keeps track of activities a member follows in the field of continuous professional development / lifelong learning. This overlaps with the variable ‘professional activities’, which is why the variable ‘registered with the KRT’ has been excluded. The year of birth of the GDP was strongly correlated with the year of graduation. The variable ‘year of birth’ of the GDP was not included as this variable was expected to have a smaller effect on the care pattern. Furthermore, a correlation was found between the variables ’GDP refers children’ and ’personal workload’. It was decided to retain the variable ’personal workload’ as Gordan et al. [27] found a relationship with restorative treatment threshold.

### Patients

The 37 participating GDPs provided data on the care provided in their dental practices to 26,216 young patients. Of these, 16,229 regular patients were included in the analyses. The mean age of these patients on 1 January 2013 was 6.8 (SD 3.5), 21.5% of them were in the age category ‘0–3’, 51.4% in the age category ‘4–9’, and 27.1% were in the age category ‘10–12’. A little more than half of the patients was boy (50.5%); 31.2% of the patients lived in a low-income neighborhood, 42.3% in a middle-income neighborhood, 25.2% in a high-income neighborhood and for 1.4% of them the income of the neighborhood was unknown. Data over 5 years were available for 79.2% of the patients. The distribution of the longitudinal preventive care patterns was as follows: 9.2% of the patients received no preventive care, 35.1% occasional preventive care, and 55.7% regular preventive care. The distribution of the longitudinal curative care patterns was: 41.6% of the patient had no curative treatments, 26.5% had curative treatment(s) in 1 year, and 31.9% curative treatments in several years.

### Preventive orientations and preventive care patterns

**Fig 1:**
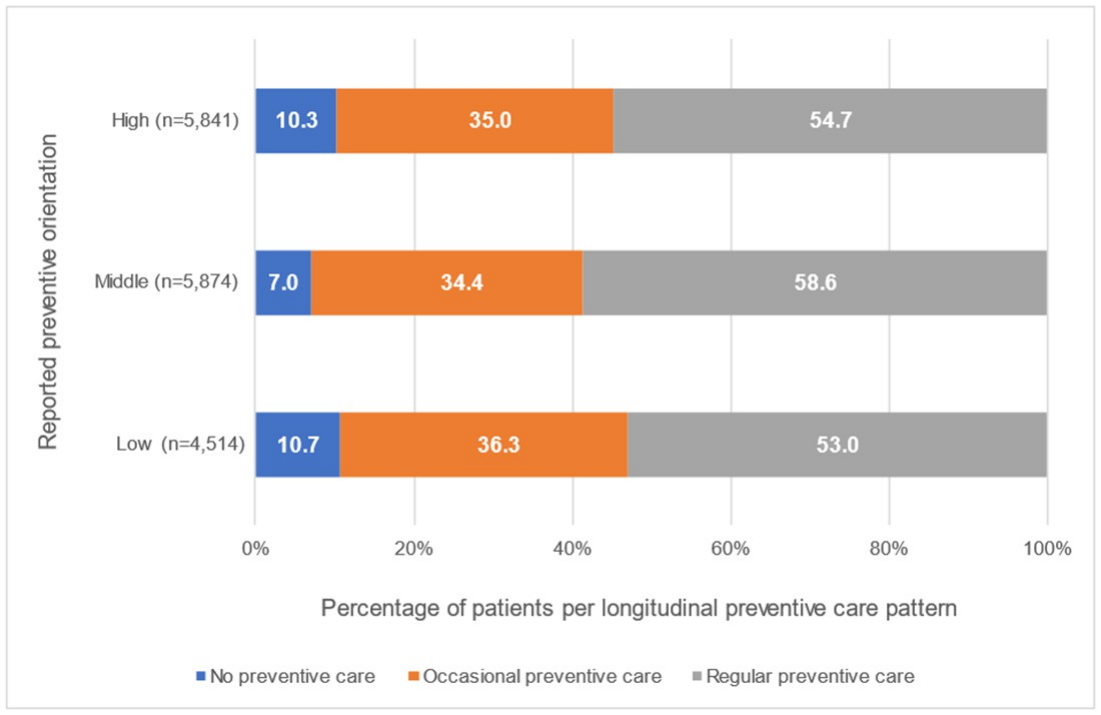
Percentage of patients per longitudinal preventive care pattern per group of GDPs with a different self-reported preventive orientation: High, middle, or low, respectively.

Fig 1 shows the percentage of patients per longitudinal preventive care pattern per group of GDPs with a different self-reported preventive orientation. There was a significant difference in the distribution of preventive care patterns between these 3 groups of patients (Kruskal Wallis test, p<0.001). The patients of GDPs who self-reported a middle preventive orientation received most often regular preventive care. The distribution of care patterns among patients of GDPs who reported a low preventive orientation and GDPs who reported a high preventive orientation was about the same.

**Fig 2:**
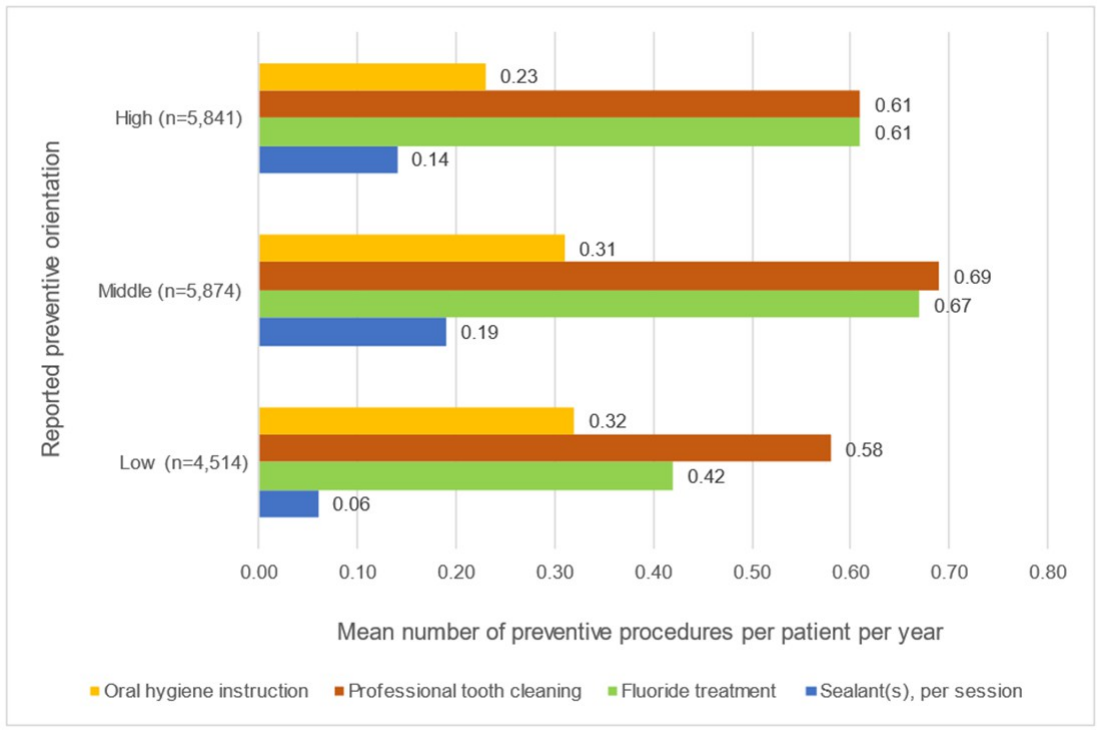
Preventive care provided per group of GDPs with a different self-reported preventive orientation: High, middle, or low, respectively.

Fig 2 shows the preventive care provided per procedure per group of GDPs with a different self-reported preventive orientation. The differences between the mean numbers of procedures per patient per year between these groups of patients were statistically significant for the mean number of oral hygiene instructions per patient per year (F(2, 16,226) = 71.179, p<0.001). Post-hoc-analyses revealed significant differences between the groups ‘low’ and ‘high’, the mean difference was 0.09 (p<0.001), and between the groups ‘middle’ and ‘high’, the mean difference between them was 0.08 (p<0.001). The difference between group ‘low’ and ‘middle’ was not statistically significant (p = 0.56). For professional tooth cleaning, the difference between the groups was statistically significant (F(2, 16,226) = 42.241, p<0.001). The mean difference between the groups ‘low’ and ‘middle’ was 0.11 (p<0.001), between the groups ‘low’ and ‘high’ 0.04 (p<0.05) and between the groups ‘middle’ and ‘high’ 0.08 (p<0.001). The difference between the groups was also statistically significant for professional fluoride applications (F(2, 16,226) = 197.212, p<0.001). The mean difference between the groups ‘low’ and ‘middle’ was 0.24 (p<0.001), between the groups ‘low’ and ‘high’ 0.18 (p<0.001) and between the groups ‘middle’ and ‘high’ 0.06 (p<0.001). Finally, the mean difference between the groups was also significant for the number of sessions concerning sealing (F(2, 16,226) = 622.156, p<0.001). The mean difference between the groups ‘low’ and ‘middle’ was 0.13 (p<0.001), between the groups ‘low’ and ‘high’ 0.08 (p<0.001) and between ‘middle’ and ‘high’ 0.05 (p<0.001). The mean number of procedures per patient per year concerning professional tooth cleaning, professional fluoride applications and sealing sessions was highest in the group with a middle preventive orientation and lowest in the group with a low preventive orientation. The patients of GDPs in group ‘high’ received the lowest number of oral hygiene instructions.

Table 2 provides the results of the multinomial logistic regression analysis (with multilevel correction for dental practice) of the relationship between the self-reported preventive orientations of GDPs and preventive care patterns, patient-, GDP-, and dental practice characteristics. Columns 2 and 3 show the descriptive information of patients, GDPs, and dental practices. Columns 4 to 6 show the parameter estimates, standard errors, and odd ratios (ORs) with the 95% confidence intervals (CI) for the outcome ‘occasional preventive care’ and columns 7 to 9 show similar information for ‘regular preventive care’. For both, the reference category was ‘no preventive care’. The results show that there was no relationship between self-reported preventive orientations and longitudinal preventive care patterns. The self-reported middle preventive orientation was neither a predictor for the care pattern ‘occasional preventive care’ (OR 1.89; 95% CI 0.52–6.82), nor for ‘regular preventive care’ (OR 12.91; 95% CI 0.76–218.65). The self-reported high preventive orientation was also neither a predictor for ‘occasional preventive care’ (OR 0.50; 95% CI 0.15–1.62), nor for ‘regular preventive care’ (OR 1.08; 95% CI 0.07–17.12).

**Table 2:** Results of the multinomial logistic regression analysis for the prediction of the preventive care pattern.

	n	n	Occasional preventive care ^a)^			Regular preventive care ^a)^		
	GDPs	patients	B ^b)^	SE	OR (95% CI)	B	SE	OR (95% CI)
*Intercept*			-0.87	0.99		**-6.24**	1.70	
Preventive orientation GDP								
*Self-reported preventive orientation*								
• low (ref)	10	4,514						
• middle	15	5,874	0.64	0.66	1.89 (0.52–6.82)	2.56	1.44	12.91 (0.76–218.65)
• high	12	5,841	-0.70	0.60	0.50 (0.15–1.62)	0.08	1.41	1.08 (0.07–17.12)
Patient characteristics								
*Age patient*								
• 0–3 (ref)		3,497						
• 4–9		8,340	**1.57**	0.24	**4.80 (2.98–7.72)**	**4.32**	0.38	**75.17 (35.40–159.62)**
• 10–12		4,392	**1.37**	0.34	**3.93 (2.04–7.58)**	**4.54**	0.44	**93.75 (39.40–223.08)**
*Gender patient*								
• boy (ref)		8,200						
• girl		8,029	-0.09	0.11	0.91 (0.73–1.14)	-0.21	0.11	0.81 (0.65–1.01)
*Average income neighborhood patient*								
• low (ref)		5,057						
middle		6,857	-0.17	0.11	0.85 (0.68–1.05)	**-0.33**	0.17	**0.72 (0.52–0.99)**
high		4,089	**-0.34**	0.14	**0.71 (0.54–0.94)**	**-0.50**	0.17	**0.61 (0.44–0.84)**
unknown		226	-0.66	0.62	0.52 (0.15–1.74)	-0.61	0.62	0.55 (0.16–1.85)
*Years included in study*								
• 5 years (ref)		12,860						
• 4 years		3,369	**-0.87**	0.16	**0.42 (0.31–0.58)**	**-1.84**	0.18	**0.16 (0.11–0.23)**
Personal characteristics GDP								
*Gender GDP*								
• male (ref)	25	10,539						
• female	12	5,690	0.84	0.64	2.32 (0.66–8.14)	1.56	1.26	4.74 (0.40–55.90)
*Year of graduation*								
• before 1995 (ref)	25	8,903						
• 1995 or later	12	7,326	0.17	1.07	1.18 (0.15–9.58)	1.19	1.55	3.29 (0.16–68.85)
*Place of graduation*								
• Amsterdam (ref)	13	6,777						
• Groningen	8	2,662	**2.95**	0.76	**19.10 (4.27–85.35)**	**5.05**	1.10	**156.32 (17.95–1361.25)**
• Nijmegen	10	4,221	**2.45**	0.52	**11.58 (4.20–31.98)**	**4.85**	1.14	**127.63 (13.55–1202.09)**
• elsewhere	6	2,569	**2.64**	0.86	**14.06 (2.61–75.66)**	**3.77**	1.46	**43.35 (2.49–755.03)**
Professional beliefs and behaviors GDP								
*Communicative style of practice*								
• no (ref)	14	8,791						
• yes	23	7,438	0.24	0.46	1.28 (0.52–3.13)	1.78	1.00	5.93 (0.83–42.29)
*Experiences an obstacle in the treatment of young children*								
• no (ref)	27	12,247						
• yes	10	3,982	**2.16**	0.74	**8.70 (2.04–37.18)**	**3.90**	1.52	**49.50 (2.51–976.57)**
*Use of clinical guidelines*								
• never/sometimes (ref)	13	4,700						
• regularly/often	24	11,529	**2.24**	0.76	**9.39 (2.12–41.54)**	1.69	1.58	5.41 (0.24–120.55)
*Use of diagnostic methods*								
• 3–6 methods (ref)	27	11,854						
• 7 methods	10	4,375	0.22	0.27	1.25 (0.74–2.13)	1.23	0.64	3.40 (0.98–11.81)
*Registration of caries risk as regular part of a ROE*								
• no (ref)	17	6,444						
• yes	20	9,785	-0.90	1.42	0.41 (0.03–6.58)	-1.19	1.84	0.31 (0.01–11.29)
*Age limit for a restorative intervention*								
• no (ref)	34	15,329						
• yes	3	900	0.69	1.38	1.98 (0.13–29.43)	1.10	1.68	2.99 (0.11–80.98)
Professional characteristics GDP								
*Owner of the dental practice*								
• no (ref)	3	1,822						
• yes	34	14,407	0.09	1.24	1.10 (0.10–12.47)	1.79	1.73	5.98 (0.20–176.63)
*Number of patients per week GDP*								
• <100 (ref)	19	8,947						
• ≥100	18	7,282	-1.01	0.82	0.37 (0.07–1.82)	-1.57	1.21	0.21 (0.02–2.25)
*Percentage of young patients GDP*								
• ≤20% (ref)	27	11,040						
• >20%	10	5,189	**-0.92**	0.46	**0.40 (0.16–0.98)**	-0.82	1.28	0.44 (0.04–5.38)
*Refers young patients (<18 yrs)*								
• no (ref)	16	8,325						
• yes	21	7,904	-0.17	0.47	0.85 (0.34–2.12)	-0.46	0.92	0.63 (0.11–3.82)
*Able to fulfill the care demand*								
• within normal working hours (ref)	32	12,977						
• by working overtime	5	3,252	**1.88**	0.45	**6.55 (2.70–15.90)**	**3.40**	0.78	**30.09 (6.51–139.16)**
*Professional activities per month*								
• ≤10 hours (ref)	17	6,431						
• >10 hours	20	9,798	0.62	0.57	1.85 (0.61–5.60)	**1.97**	1.00	**7.20 (1.01–51.48)**
Characteristics dental practice								
*Number of patients dental practice*								
• ≤2000 (ref)	15	3,566						
• 2001–4000	16	7,658	**-1.85**	0.58	**0.16 (0.05–0.49)**	**-3.23**	1.01	**0.04 (0.01–0.29)**
• >4000	6	5,005	0.08	0.91	1.09 (0.18–6.48)	0.16	1.36	1.17 (0.08–16.84)
*Percentage of young patients dental practice*								
• ≤20% (ref)	28	11,834						
• >20%	9	4,395	**2.80**	0.94	**16.52 (2.62–104.01)**	**4.59**	1.42	**98.50 (6.06–1600.60)**
*Task executed by oral hygienist and/or prevention assistant*								
• 0 (ref)	5	1,651						
• 1 to 4	15	5,504	0.04	0.37	1.04 (0.51–2.14)	-1.48	0.96	0.23 (0.04–1.49)
• 5 to 8	17	9,074	0.44	0.52	1.56 (0.56–4.29)	-1.87	1.09	0.16 (0.02–1.31)
*Practice policy on provision of care to young patients*								
• no (ref)	15	4,915						
• yes	22	11,314	**1.53**	0.63	**4.63 (1.35–15.85)**	**2.34**	1.08	**10.34 (1.26–84.94)**
*Inhabitants per GDP in region*								
• <1800 (ref)	7	2,144						
• 1800–2200	10	4,385	**-1.26**	0.52	**0.28 (0.10–0.79)**	-1.82	1.68	0.16 (0.01–4.39)
• >2200	20	9,700	**-2.70**	1.08	**0.07 (0.01–0.56)**	**-3.80**	1.67	**0.02 (0.00–0.58)**

^a)^ The reference category was no preventive care

^b)^ Bold means a significant difference (p<0.05)

The results were corrected for dental practice by a multilevel approach. Columns 2 and 3 show the descriptive information of patients, GDPs, and dental practices. Columns 4 to 6 show the parameter estimates, standard errors, and odd ratios (OR) with the 95% confidence intervals (95% CI) for the outcome ‘occasional preventive care’ and columns 7 to 9 show similar information for ‘regular preventive care’. For both, the reference category was ‘no preventive care’.

The odds ratios (OR) were the ratio of the probability of the presence of a certain outcome (occasional preventive care, respectively regular preventive care) over the probability of its absence (the reference category no preventive care). First, the multinomial logistic regression analysis was done to explore the effects of all predictors on occasional preventive care versus no preventive care and then on regular preventive care versus no preventive care. Only the significant predictors are named in the results, the ORs and 95% CIs for all predictors are shown in Table 2.

The OR for age 4–9 compared to 0–3 was 4.80 (95% CI 2.98–7.72) for occasional preventive care versus no preventive care. This implies that compared to toddlers (age 0–3) the probability of getting occasional preventive care was higher for patients who were 4–9 years old at the beginning of the study period. The same holds for patients aged 10–12 (OR 3.93; 95% CI 2.04–7.58). The probability of occasional preventive versus no preventive care was also significantly higher if the GDP was graduated in another place than Amsterdam, experienced an obstacle in the treatment of young children, used clinical guidelines regularly/often, was able to fulfill the care demand by working overtime. It was also higher if the dental practice had >20% young patients, a practice policy on the provision of care to young patients, and was in a region with a high dentist ratio (<1,800 inhabitants per GDP). The probability of getting occasional preventive care was lower if the patient was from a high-income neighborhood, and was included in the study for 4 years, if the GDP had ≤20% young patients, and if the dental practice was middle sized (2001–4000 patients).

The probability of regular preventive care versus no preventive care was significantly higher if the patient was 4–9 or 10–12 years old at the beginning of the study. Also, if the GDP was graduated in another place than Amsterdam, experienced an obstacle in the treatment of young children, was able to fulfill the care demand by working overtime, and spent more than 10 hours per month on professional activities. It was also higher if the dental practice had >20% young patients, and a practice policy on the provision of care to young patients. The probability of getting regular preventive care was lower if the patient was from a middle- or high-income neighborhood, and was included in the study for 4 years, if the dental practice was middle sized (2001–4000 patients), and in a region with a low dentist ratio (>2,200 inhabitants per GDP).

### Curative orientations and curative care patterns

**Fig 3:**
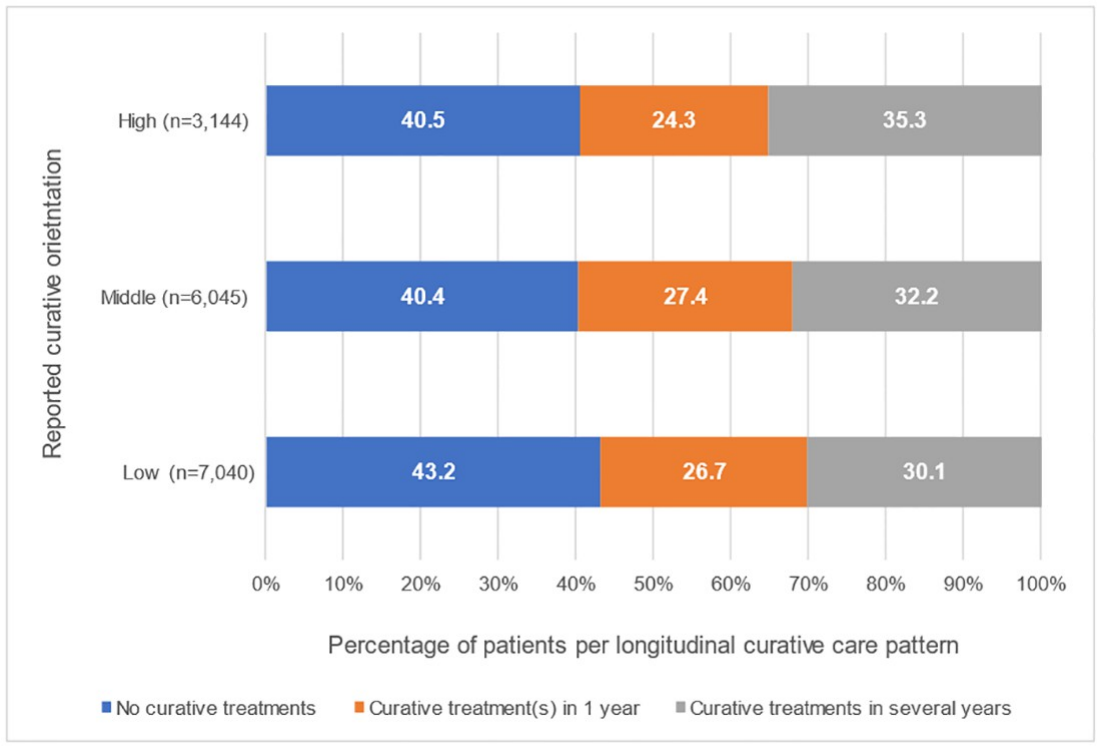
Percentage of patients per longitudinal curative care pattern per group of GDPs with a different self-reported curative orientation: High, middle, or low, respectively.

Fig 3 shows the percentage of patients per longitudinal curative care pattern per group of GDPs with a different self-reported curative orientation. There was a significant difference in the distribution of curative care patterns between these 3 groups of patients (Kruskal Wallis test, p<0.001). Among the GDPs who reported a low curative orientation, the highest percentage of patients did not get any curative treatment. The higher the category for self-reported curative orientation, the higher the percentage of patients with curative treatments in several years.

**Fig 4:**
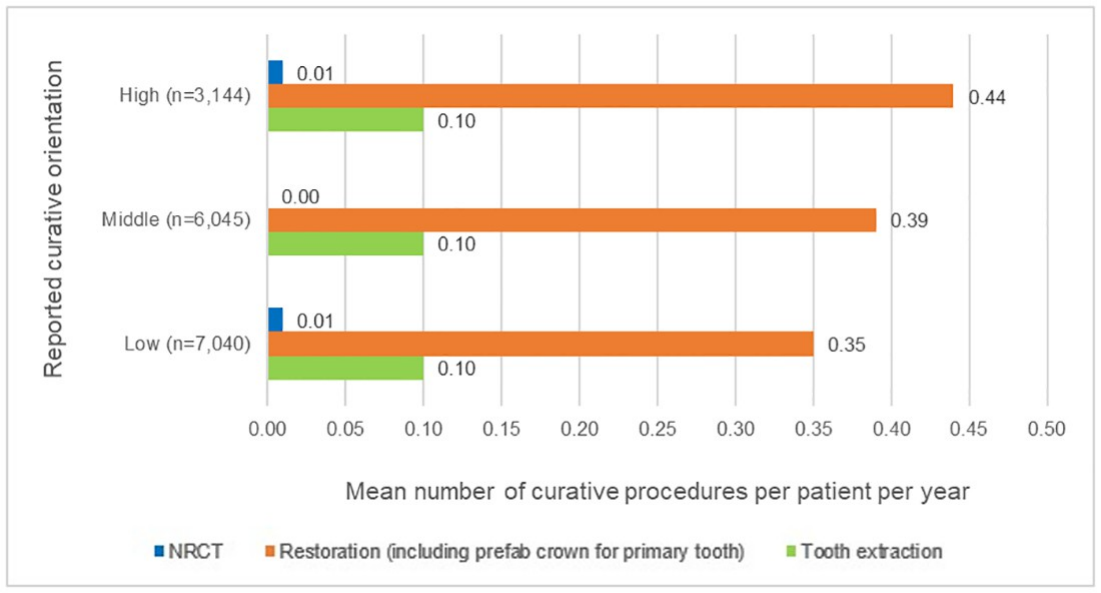
Curative care provided per group of GDPs with a different self-reported curative orientation: High, middle, or low, respectively.

Fig 4 shows the curative care provided per procedure per group of GDPs with a different self-reported curative orientation. Patients of GDPs with a different curative orientation differed especially in the mean number of restorations per year. The higher the category of curative orientation, the higher the mean number of restorations per patient per year. These differences were statistically significant (F(2, 16,226) = 21.986, p<0.001). The post hoc test revealed that the mean difference between the groups ‘low’ and ‘middle’ was 0.04 (p<0.001), between groups ‘low’ and ‘high’ 0.09 (p<0.001), and between ‘middle’ and ‘high’ 0.05 (p<0.01).

Table 3 provides the results of the multinomial logistic regression analysis (with a multilevel correction for dental practice) of the relationship between the self-reported curative orientations of GDPs and curative care patterns, patient-, GDP-, and dental practice characteristics. The reference category for curative care patterns was no curative care. The results showed a relationship between self-reported curative orientations and longitudinal curative care patterns. The self-reported middle curative orientation was a predictor of the care pattern ‘curative treatment(s) in 1 year’ (OR 1.23; 95% CI 1.02–1.48). The self-reported high curative orientation was a predictor of ‘curative treatments in several years’ (OR 1.90; 95% CI 1.27–2.85).

**Table 3:** Results of the multinomial logistic regression analysis for the prediction of the curative care pattern.

			Curative treatment(s) in 1 year ^a)^			Curative treatments in several years ^a)^		
	n GDPs	n patients	B ^b)^	SE	OR (95% CI)	B	SE	OR (95% CI)
*Intercept*			**-0.69**	0.16		**-1.24**	0.35	
Curative orientation GDP								
*Self-reported curative orientation*								
• low (ref)	17	7,040						
• middle	12	6,045	**0.20**	0.10	**1.23 (1.02–1.48)**	0.28	0.18	1.32 (0.93–1.88)
• high	8	3,144	0.16	0.12	1.17 (0.93–1.48)	**0.64**	0.21	**1.90 (1.27–2.85)**
Patient characteristics								
*Age patient*								
• 0–3 (ref)		3,497						
• 4–9		8,340	**0.69**	0.05	**2.00 (1.80–2.21)**	**0.97**	0.06	**2.65 (2.35–2.99)**
• 10–12		4,392	**0.86**	0.08	**2.36 (2.04–2.73)**	**0.86**	0.07	**2.36 (2.06–2.70)**
*Gender patient*								
• boy (ref)		8,200						
• girl		8,029	0.05	0.03	1.06 (0.99–1.12)	0.01	0.05	1.01 (0.92–1.11)
*Average income neighborhood patient*								
• low (ref)		5,057						
• middle		6,857	**-0.23**	0.07	**0.79 (0.70–0.90)**	**-0.42**	0.07	**0.66 (0.57–0.75)**
• high		4,089	**-0.28**	0.07	**0.76 (0.66–0.87)**	**-0.57**	0.08	**0.57 (0.49–0.66)**
• unknown		226	-0.08	0.17	0.92 (0.66–1.29)	-0.10	0.18	0.91 (0.64–1.30)
*Years included in study*								
• 5 years (ref)		12,860						
• 4 years		3,369	**-0.10**	0.04	**0.91 (0.83–0.98)**	**-0.38**	0.05	**0.68 (0.62–0.75)**
Personal characteristics GDP								
*Gender GDP*								
• male (ref)	25	10,539						
• female	12	5,690	0.12	0.20	1.13 (0.76–1.67)	0.49	0.34	1.63 (0.83–3.20)
*Year of graduation*								
• before 1995 (ref)	25	8,903						
• 1995 or later	12	7,326	-0.09	0.15	0.91 (0.68–1.23)	-0.00	0.27	1.00 (0.59–1.68
*Place of graduation*								
• Amsterdam (ref)	13	6,777						
• Groningen	8	2,662	-0.02	0.12	0.98 (0.77–1.25)	0.37	0.23	1.45 (0.92–2.28)
• Nijmegen	10	4,221	0.05	0.12	1.05 (0.83–1.32)	-0.11	0.21	0.90 (0.60–1.35)
• elsewhere	6	2,569	-0.21	0.12	0.81 (0.65–1.02)	-0.30	0.24	0.74 (0.46–1.18)
Caries-related opinions GDP								
*Estimated lesion progression rate*								
• lower than average (ref)	17	8,557						
• higher than average	20	7,672	**0.22**	0.09	**1.25 (1.05–1.47)**	**0.49**	0.17	**1.64 (1.18–2.27)**
*Estimated percentage cavitated lesions*								
• lower than average (ref)	20	9,200						
• higher than average	17	7,029	0.14	0.13	1.15 (0.88–1.49)	0.42	0.25	1.52 (0.93–2.48)
*Depth of a radiographic lesion is an underestimate*								
• no (ref)	8	4,221						
• yes	29	12,008	**-0.35**	0.09	**0.70 (0.59–0.84)**	**-0.42**	0.19	**0.66 (0.45–0.95)**
Professional beliefs and behaviors GDP								
*Communicative style of practice*								
• no (ref)	14	8,791						
• yes	23	7,438	**-0.26**	0.12	**0.77 (0.62–0.97)**	**-0.51**	0.20	**0.60 (0.40–0.89)**
*Use of clinical guidelines*								
• never/sometimes (ref)	13	4,700						
• regularly/often	24	11,529	**0.49**	0.13	**1.63 (1.25–2.11)**	**1.13**	0.22	**3.09 (2.01–4.73)**
*Use of diagnostic methods*								
• 3–6 methods (ref)	27	11,854						
• 7 methods	10	4,375	**-0.17**	0.07	**0.85 (0.74–0.97)**	-0.02	0.13	0.98 (0.76–1.26)
*Registration of caries risk as regular part of a ROE*								
• no (ref)	17	6,444						
• yes	20	9,785	**-0.51**	0.15	**0.60 (0.45–0.81)**	**-1.23**	0.24	**0.29 (0.18–0.47)**
*Age first ROE*								
• between 0 and 2 yrs (ref)	18	8,165						
• between 2 and 4 yrs	19	8,064	0.02	0.15	1.02 (0.75–1.37)	-0.12	0.28	0.89 (0.52–1.53)
Professional characteristics GDP								
*Owner of the dental practice*								
• no (ref)	3	1,822						
• yes	34	14,407	0.14	0.12	1.15 (0.91–1.46)	0.39	0.25	1.48 (0.92–2.39)
*Percentage of young patients GDP*								
• ≤20% (ref)	27	11,040						
• >20%	10	5,189	0.19	0.21	1.21 (0.79–1.83)	-0.11	0.37	0.90 (0.44–1.84)
*Refers young patients (<18 yrs)*								
• no (ref)	16	8,325						
• yes	21	7,904	-0.11	0.11	0.90 (0.72–1.11)	-0.26	0.19	0.77 (0.53–1.13)
*Able to fulfill the care demand*								
• within normal working hours (ref)	32	12,977						
• by working overtime	5	3,252	**0.31**	0.12	**1.36 (1.08–1.72)**	**0.84**	0.25	**2.31 (1.41–3.76)**
*Professional activities per month*								
• ≤10 hours (ref)	17	6,431						
• >10 hours	20	9,798	0.07	0.06	1.07 (0.96–1.19)	0.16	0.12	1.18 (0.94–1.48)
Characteristics dental practice								
*Number of patients dental practice*								
• ≤2000 (ref)	15	3,566						
• 2001–4000	16	7,658	**-0.54**	0.26	**0.58 (0.35–0.96)**	**-1.24**	0.46	**0.29 (0.12–0.72)**
• >4000	6	5,005	-0.37	0.22	0.69 (0.45–1.05)	**-1.09**	0.39	**0.34 (0.16–0.72)**
*Practice policy on the provision of care to young patients*								
• no (ref)	15	4,915						
• yes	22	11,314	**0.36**	0.15	**1.43 (1.06–1.92)**	**1.20**	0.24	**3.31 (2.09–5.24)**
*Inhabitants per GDP in region*								
• <1800 (ref)	7	2,144						
• 1800–2200	10	4,385	-0.30	0.19	0.74 (0.51–1.07)	-0.13	0.32	0.88 (0.47–1.66)
• >2200	20	9,700	-0.10	0.13	0.91 (0.70–1.18)	0.07	0.23	1.07 (0.68–1.70)

^a)^ The reference category was no curative treatments

^b)^ Bold means a significant difference (p<0.05)

The results were corrected for dental practice by a multilevel approach. Columns 2 and 3 show the descriptive information of patients, GDPs, and dental practices. Columns 4 to 6 show the parameter estimates, standard errors, and odd ratios (OR) with the 95% confidence intervals (95% CI) for the outcome ‘curative treatment(s) in 1 year’ and columns 7 to 9 show similar information for ‘curative treatments in several years’. For both, the reference category was ‘no curative treatments’.

The effects of all predictors are shown in Table 3, but only the significant effects are described below. The probability of curative treatment(s) in 1 year versus no curative treatments was significantly higher if the patient was 4–9 or 10–12 years old at the beginning of the study. This was also found if the GDP estimated the rate of lesion progression to be higher than average, thought that the depth of a radiographic lesion is not an underestimate of the actual depth, recognized him- or herself mostly in another style of practice than communicative, used clinical guidelines regularly/often, used 3–6 diagnostic methods, could fulfill the care demand by working overtime. It was also higher if the dental practice had a practice policy on the provision of care to young patients. The probability of getting curative treatment(s) was lower if the patient was from a middle- or high-income neighborhood, and was included in the study for 4 years, if the GDP recorded the caries risk of a patient as regular part of a ROE, and if the dental practice was middle sized (2001–4000 patients).

The probability of curative treatments in several years versus no curative treatments was significantly higher if the patient was 4–9 or 10–12 years old at the beginning of the study. This was also found if the GDP estimated the rate of lesion progression to be higher than average, thought that the depth of a radiographic lesion is not an underestimate of the actual depth, recognized him- or herself mostly in another style of practice than communicative, used clinical guidelines regularly/often, could fulfill the care demand by working overtime. It was also higher if the dental practice was small (≤2,000 patients), and had a practice policy on the provision of care to young patients. The probability of getting curative treatments in several years was lower if the patient was from a middle- or high-income neighborhood, and was included in the study for 4 years, and if the GDP recorded the caries risk of a patient as regular part of a ROE.

## Discussion

In this study, a relationship was found between the self-reported curative orientation and provided curative care, but not between the self-reported preventive orientation and provided preventive care. GDPs with a self-reported middle preventive orientation provided most preventive care. There were several patient-, GDP- and dental practice characteristics related to preventive and curative care patterns. Supplementary file S8 Table provides a summary of our findings. Some unexpected or remarkable findings are highlighted here. Firstly, the rate of lesion progression was overestimated. All but 1 GDP thought that it would take less than 24 months for a lesion to progress from the ‘enamel-dentin border’ to the ‘inner third of the dentin’ in a 15-year-old. According to Mejàre et al. [28], the median survival time of lesions with a ‘broken enamel-dentin border but with no obvious progression in the dentin’ to ‘obvious spread in the outer half of the dentin’ is already about 37 months (3.1 years with a range from 2.0 to 6.8 years) in 11- to 22-year-olds. GDPs who estimated the rate of lesion progression to be higher than average provided more curative procedures. This may suggest that some patients have been overtreated. Less curative treatments were done by GDPs who registered the caries risk of a patient as regular part of a ROE. The decision to intervene curatively was probably made more on an individual patient basis by them. Secondly, some GDPs were active in both preventive care and curative treatments. Common characteristics predicting extensive care (regular preventive care and curative treatments in several years) were patient related: age category 4–9, age category 10–12, from low-income neighborhood, 5 years included in study. But there were also GDP and dental practice related predictors: the GDP can fulfill the care demand by working overtime, small dental practice (≤2,000 patients), and practice policy on the provision of care to young patients. This strongly suggests that the provided care was at least partly supplier-driven and workload related, instead of individually indicated, which is in line with previous findings of Signori et al. [2]. Thirdly, GDPs who reported using clinical guidelines regularly or often provided more curative treatments and given our results therefore probably intervened in an earlier stage of the caries process. This provision of restorative care is not in line with current evidence and recommendations to monitor lesion progression and thus wait longer with restorative interventions. This corroborated findings by Schwendicke et al. [29] who reported that the most up-to-date knowledge in the field of caries prevention and treatment is only slowly implemented into clinical practice.

The comparison between the answers to the questionnaire and the provided OHS has four possible interpretation issues. Firstly, there was some time between completing the questionnaire (2020) and the period in which the care was provided (2013–2017). Vertimart had already built in the application in Exquise for the automatic data extraction for the period 2013–2017. Then, the recruitment of participants was delayed due to circumstances including the Covid pandemic. In the meantime, the caries management approach of a GDP could have changed. In fact, this was mentioned by a participant. Around the time of filling in the questionnaire, the clinical practice guideline Oral healthcare (0–18 yrs)—prevention and treatment of dental caries (2020) has been released. Also, there is a tendency for some years towards a higher preventive orientation as an increasing number of dental practices have implemented a prevention program based on the Nexø model (Gewoon Gaaf). In 2022, this applied to 11% of the Dutch dental practices [30]. As a result, the self-reported preventive orientation may have been overestimated. Socially desirable responding could also have played a role. Nonetheless, it was also possible that GDPs with a high preventive orientation had a patient population that no longer needed much preventive care. Secondly, NRCT was not reimbursed under the standard healthcare insurance and may have been claimed as curative treatment (restoration), or as prevention (oral hygiene instruction and/or professional fluoride application). The latter may have led to an underestimation of the provided curative treatments. Thirdly, multiple caries management options could be chosen for a particular clinical case in the questionnaire. In some cases, the given answers were incompatible like both restoration and tooth extraction. Some GDPs probably indicated the caries management options they would choose from in a particular situation. Fourthly, the questionnaires were completed by the participants and the OHS were provided by all workers in the dental practice. Nevertheless, it can be expected that the care provided by the dental practice had sufficient overlap with the care provided by the participants as three-quarters of the participants reported working in a dental practice with a practice policy on the provision of care to young patients.

The answers to the questionnaire provided a picture of the extent to which the self-reported caries management approaches and opinions of GDPs were in line with current clinical guidelines. That was not always the case. More than half of the participating GDPs said they would start with a ROE when children are between 2 and 4 years old. The clinical practice guideline Oral healthcare (0–18 yrs)—prevention and treatment of dental caries [4] recommends that this should be started shortly before or just after the eruption of the first tooth, so approximately around 6 months. Only two participants would do this. The same guideline states that sealants and professional fluoride applications should not be provided routinely if there is no caries activity. Fluoride applications were given on a large scale. More than 40% of the patients did not get any curative treatment, but 60% of the patients received fluoride applications with an average of 1 treatment per year. It is plausible to suppose that some of them might be seen as overtreatment. In case of caries in an occlusal surface of a permanent tooth, most of the participating GDPs (78% in a hypothetical low-risk-, and 97% in a high-risk patient) would intervene restoratively at stage 3 (lesion to the outer third of dentin) or earlier. In case of caries in an approximal surface of a permanent tooth, many GDPs would also intervene restoratively at stage 3 or earlier (57% in a hypothetical low-risk-, and 97% in a high-risk patient). Nowadays, postponing restorative treatment until a caries lesion is cavitated should be seen as contemporary practice [15, 31]. Then, the restorative treatment threshold should be closer to stage 4 (lesion up to the middle third of dentin) [32]. Intervening too early is undesirable as it starts the re-restoration cycle [33]. A caries lesion will progress if the process is not influenced. However, until a considerable time in the caries process, preventive care and proper self-care may stop or slow down the progression of a caries lesion, leaving an arrested lesion [34]. If that fails and the caries process continues, the chance of cavitation increases. Restorative intervention before the lesion is cavitated leads to the restoration cycle being started too early, which ultimately increases the chance of losing the tooth. Restorations must be replaced from time to time leaving less and less of the tooth structure [17].

### Strengths and limitations

A strength of this study was the availability of longitudinal data for a large number of patients, and the comparison of self-reported orientations of GDPs to the care provided to all of their regular patients under 18 years old. To the best of our knowledge, this has not been done before. Previous studies looked at actual restoration depths in patient records [16, 17].

A limitation of this study was the low number of participating GDPs. Compared to all Dutch dentists, males and practice owners were overrepresented among the participants. They also graduated relatively long ago. The participants were more than average focused on professional activities, 80% were registered with the KRT compared to 52% nationally [35] and an average of about 13 hours per month was spent on professional activities compared to about 10 nationally [23]. Nonetheless, there is no reason to assume that the results do not reflect the usual situation in the Netherlands.

### Relevance

In the late 1980s den Dekker [22] researched treatment planning in dental practices and found a great deal of variation in the treatment plans proposed by GDPs. At the beginning of this century, Bruers [11] again found differences between GDPs in the care provided to (comparable) patients. Nowadays, almost 20 years later, little seems to have changed. After correction for patient characteristics, the provision of more extensive OHS was related to several GDP and dental practice characteristics. Thus, instead of being evidence-based, the provided OHS was at least partly supply-driven [36]. It is harmful for the oral health of patients if a restorative intervention is carried out too early. It is detrimental to the affordability of care if more preventive care and curative treatments are given than is required according to evidence based guidance. In the Netherlands, many dental practices provide frequent oral hygiene instructions and sessions concerning professional tooth cleaning to a large part of their young patients. However, this care is only cost effective if it adds to the oral health of a patient. That is the case if a patient has an elevated or high caries risk, and the patient and/or its parents are motivated and able to change their behavior. Professional preventive care is not intended to compensate or take over daily oral care. To prevent overtreatment, some healthcare insurance companies have therefore introduced an authorization requirement for preventive care exceeding certain limits. Clinical guidelines can provide guidance on appropriate care, but dissemination and finally their implementation in dental practice does not happen automatically [37]. Dental schools and providers of postgraduate education should play an important role in this quality process. KIMO is developing clinical practice guidelines but so far has no resources to pay attention to the implementation process. Nevertheless, a set of indicators for the use and adherence is added to each guideline. In general, such indicators concern counts for how often a certain procedure has been provided, for example the average number of restorations per 100 children [4]. Our study showed that this cannot be related directly to health outcomes. GDPs with similar patients in terms of caries activity, but with different restorative treatment thresholds, claimed different numbers of restorations. To achieve quality improvement, it is desirable that GDPs become more aware of differences in their treatment approaches and discuss them [38]. The combination of answers to hypothetical clinical situations and benchmark information on provided care, as we did in this study, can give focus and depth to such discussions.

## Conclusions

There was a relationship between the self-reported curative orientation and provided curative care, but not between the self-reported preventive orientation and provided preventive care. GDPs with a self-reported middle preventive orientation provided most preventive care. So, the provision of preventive care was probably not related to clinical situations of patients. The rate of lesion progression was overestimated, which makes that most of the participating GDPs would probably intervene restoratively earlier in the caries process than is recommended nowadays. Given the relationship between the self-reported curative orientation and provided curative care, this may have led to overtreatment.

After correction for patient characteristics, provided preventive and curative care patterns were related to several GDP and dental practice characteristics. This revealed that the provided care was, at least partly, supplier-driven instead of patient-centered leading to unwarranted treatment variation. Effort is needed to stimulate appropriate, person-centered oral health care. That is, do enough to prevent oral diseases, and avert iatrogenic damage and unnecessary higher costs.

## Supporting information

S1 File (DOCX)

S1 Table General personal and professional characteristics participating general dental practitioners (GDPs). (DOCX)

S2 Table Opinions of general dental practitioners (GDPs) on the management of dental caries in the primary dentition. (DOCX)

S3 Table Opinions of general dental practitioners (GDPs) on the management of dental caries in an occlusal surface of a permanent tooth. (DOCX)

S4 Table Opinions of general dental practitioners (GDPs) on the management of dental caries in an approximal surface of a permanent tooth. (DOCX)

S5 Table Estimated severity of caries lesions by general dental practitioners (GDPs). A. Lesion progression in an approximal surface of a permanent tooth in a 15-year-old patient without orthodontic braces. Estimated time needed for progression from the first into the second stage. B. Estimated percentage of cavitated lesions in different stages of the caries process. C. Estimation of the relationship between the actual depth of a caries lesion and the image on a bitewing radiograph.
(DOCX)

S6 Table Professional opinions and behaviors of the participating general dental practitioners (GDPs). (DOCX)

S7 Table Characteristics of the participating dental practices. (DOCX)

S8 Table Variables predicting preventive and curative care patterns (based on p-value <0.05). (DOCX)

## Abbreviations

GDP: general dental practitioner
KNMT: Royal Dutch Dental Association
NRCT: non-restorative cavity treatment
OHS: oral healthcare services
ROE: routine oral health examination
