# Supplementary material for: The relationship between self-reported preventive and curative orientations of dentists and oral healthcare services provided to Dutch young patients: An observational study
Source: PLoS One. 2024 Jul 5;19(7):e0306403. doi: 10.1371/journal.pone.0306403 (PMC11226104; doi:10.1371/journal.pone.0306403)
Supplement: S1 File — (DOCX) [file pone.0306403.s001.docx]

**S1 Questionnaire.**

**Instruction**

This questionnaire is about your assessment of a number of clinical situations concerning the management of dental caries in children. Then, you will be asked about some characteristics of your dental practice and work situation.

For most questions you are expected to give one answer. For several other questions, multiple answers are possible. If this is the case, this will be stated in the question. For some questions you will be asked to write an answer yourself. Your dental practice refers to the dental practice with which you participate in this study.

**Clinical cases**

*In the following questions you will be asked about your treatment approach in various clinical situations and your assessment of the severity of the situation.*

| 1 | Different stages of caries progression in primary teeth are shown below.  Would you please indicate what your treatment approach would be for each stage?  *Multiple answers are possible per stage.* | | | | |
| --- | --- | --- | --- | --- | --- |
| *^a)^* | | 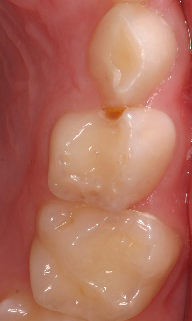 | 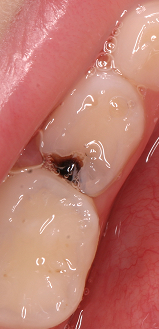 | 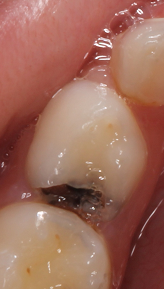 | 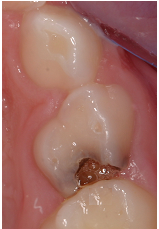 |
| *^b)^* | | *Stage 1*  *A mesial occlusal cavity affecting less than half of the marginal ridge in the upper first primary molar tooth. The tooth is vital, and the child has no history of pain* | *Stage 2*  *A disto occlusal cavity affecting more than half of the marginal ridge in the lower first primary molar tooth. The tooth is vital, and the child has no history of pain.* | *Stage 3*  *A large distal occlusal cavity in the lower first primary molar tooth affecting more than half of the marginal ridge. The tooth is non-vital, but the child has no history of pain.* | *Stage 4*  *A large distal occlusal cavity in the upper first primary molar where more than half of the marginal ridge has been lost. The child is experiencing pain and has a swollen cheek.* |
|  | Each stage refers to a 6-year-old patient with one cavity. The child sits quietly in the chair and can be looked into the mouth without any problems. | | | | |
| a | Monitoring |  |  |  |  |
| b | Prescribing/advi-sing a painkiller |  |  |  |  |
| c | Prescribing antibiotics |  |  |  |  |
| d | Oral hygiene instruction |  |  |  |  |
| e | Professional fluoride application |  |  |  |  |
| f | Non-restorative cavity treatment |  |  |  |  |
| g | ART-restoration |  |  |  |  |
| h | Restoration |  |  |  |  |
| g | Prefab crown/ Hall technique |  |  |  |  |
| i | Opening the pulp chamber and allowing the tooth to drain |  |  |  |  |
| j | Pulpotomy |  |  |  |  |
| k | Tooth extraction |  |  |  |  |
| l | Refer for tooth extraction |  |  |  |  |
| *^a)^ The photographs of the caries lesions were provided by the department of pediatric dentistry of ACTA.*  *^b)^ The descriptions were reused with minor adjustments from* *Tickle M, Threlfall AG, Pilkington L, Milsom KM, Duggal MS, Blinkhorn AS. Approaches taken to the treatment of young children with carious primary teeth: a national cross-sectional survey of general dental practitioners and paediatric specialists in England. Br Dent J. 2007 Jul28; 203(2): E4; discussion 102-103.* | | | | | |

| 2a | The photographs below show different stages of caries progression in the occlusal surface in a permanent tooth. Would you please indicate for each stage what your treatment approach would be?  *Multiple options are possible per stage.* | | | | | | | |
| --- | --- | --- | --- | --- | --- | --- | --- | --- |
| *^a)^* | | 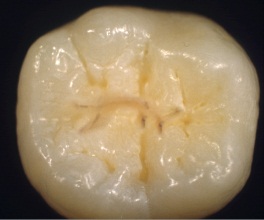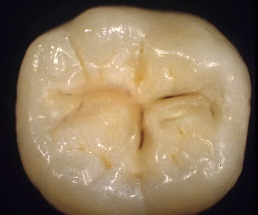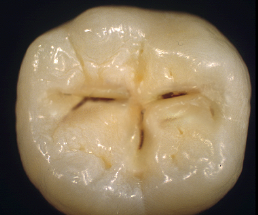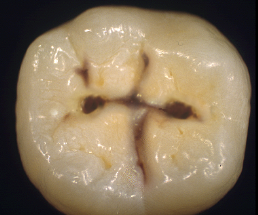 | | | | | | |
| *^b)^* | | *Stage I*   - *White / brownish discoloration in the enamel, no signs of cavitation.* - *No radiographic signs of caries.* | *Stage II*   - *Minor loss of tooth substance, with caries in the enamel.* - *No radiographic signs of caries.* | | *Stage III*   - *Moderate loss of tooth substance.* - *Caries in the outer third of the dentin according to the radiograph.* | | *Stage IV*   - *Considerable loss of tooth substance.* - *Caries up to the middle third of the dentin according to the radiograph.* | |
| I | Case I  A 15-year-old patient without orthodontic appliances. The patient has low caries activity and good oral hygiene, has been attending the dental practice regularly for routine oral examinations, and claims to brush twice a day with a fluoridated toothpaste. | | | | | | | |
| a | Monitoring |  | |  | |  | |  |
| b | Oral hygiene instruction |  | |  | |  | |  |
| c | Professional fluoride application |  | |  | |  | |  |
| d | Sealant |  | |  | |  | |  |
| e | Restoration |  | |  | |  | |  |
| f | Other, namely…  *(please fill in)* |  | |  | |  | |  |
| *^a)^ The photographs were reused from: Espelid I, Tveit AB, Mejàre I, Sundberg H, Hallonsten AL. Restorative treatment decisions on occlusal caries in Scandinavia. Acta Odontol Scand. 2001; 59: 21–27.*  *^b)^ The descriptions were reused from: Mejàre I, Sundberg H, Espelid I, Tveit B. Caries assessment and restorative treatment thresholds reported by Swedish dentists. Acta Odontol Scand. 1999; 57: 149–154. Mejàre et al. (1999).* | | | | | | | | |

| 2b | The photographs below show different stages of caries progression in the occlusal surface in a permanent tooth. Would you please indicate for each stage what your treatment approach would be?  *Multiple options are possible per stage.* | | | | | | |
| --- | --- | --- | --- | --- | --- | --- | --- |
| *^a)^* | | 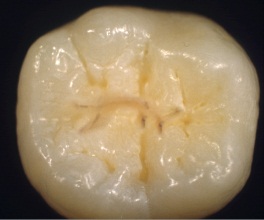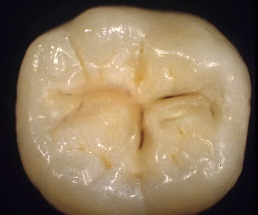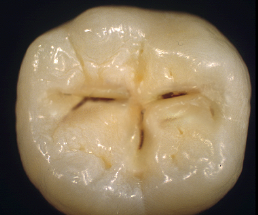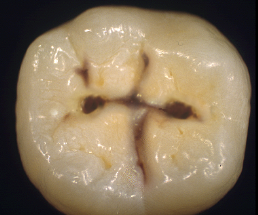 | | | | | |
| *^b)^* | | *Stage I*   - *White / brownish discoloration in the enamel, no signs of cavitation.* - *No radiographic signs of caries.* | *Stage II*   - *Minor loss of tooth substance, with caries in the enamel.* - *No radiographic signs of caries.* | *Stage III*   - *Moderate loss of tooth substance.* - *Caries in the outer third of the dentin according to the radiograph.* | | *Stage IV*   - *Considerable loss of tooth substance.* - *Caries up to the middle third of the dentin according to the radiograph.* | |
| II | Case II  A 15-year-old patient without orthodontic appliances. The patient has high caries activity and inadequate oral hygiene, has been attending the dental practice irregularly for routine oral examinations, and claims to brush once a day with a fluoridated toothpaste. | | | | | | |
| a | Monitoring |  |  | |  | |  |
| b | Oral hygiene instruction |  |  | |  | |  |
| c | Professional fluoride application |  |  | |  | |  |
| d | Sealant |  |  | |  | |  |
| e | Restoration |  |  | |  | |  |
| f | Other, namely…  *(please fill in)* |  |  | |  | |  |
| *^a)^ The photographs were reused from: Espelid I, Tveit AB, Mejàre I, Sundberg H, Hallonsten AL. Restorative treatment decisions on occlusal caries in Scandinavia. Acta Odontol Scand. 2001; 59: 21–27.*  *^b)^ The descriptions were reused from: Mejàre I, Sundberg H, Espelid I, Tveit B. Caries assessment and restorative treatment thresholds reported by Swedish dentists. Acta Odontol Scand. 1999; 57: 149–154. Mejàre et al. (1999).* | | | | | | | |

| 3a | The figures below show different stages of caries progression in the distal surface of tooth 46 at the contact point. Would you please indicate for each stage what your treatment approach would be?  *Multiple options are possible per stage.* | | | | |
| --- | --- | --- | --- | --- | --- |
| *^a)^* | | 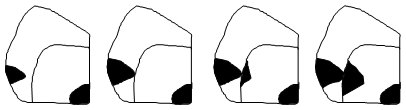 | | | |
|  | | *Stage I*  *Inner half of the enamel.* | *Stage II*  *Enamel-dentin border reached.* | *Stage III*  *Outer third of the dentin.* | *Stage IV*  *Middle third of the dentin.* |
| I. | Case I  A 15-year-old patient without orthodontic appliances. The patient has low caries activity and good oral hygiene, has been attending the dental practice regularly for routine oral examinations, and claims to brush twice a day with a fluoridated toothpaste. | | | | |
| a | Monitoring |  |  |  |  |
| b | Oral hygiene instruction |  |  |  |  |
| c | Professional fluoride application |  |  |  |  |
| d | Restoration |  |  |  |  |
| e | Other, namely…  *(please fill in)* |  |  |  |  |
| *^a)^ The figures were reused from: Mejàre I, Sundberg H, Espelid I, Tveit B. Caries assessment and restorative treatment thresholds reported by Swedish dentists. Acta Odontol Scand. 1999; 57: 149–154. Mejàre et al. (1999).* | | | | | |

| 3b | The figures below show different stages of caries progression in the distal surface of tooth 46 at the contact point. Would you please indicate for each stage what your treatment approach would be?  *Multiple options are possible per stage.* | | | | |
| --- | --- | --- | --- | --- | --- |
| *^a)^* | | 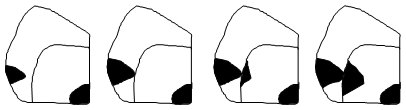 | | | |
|  | | *Stage I*  *Inner half of the enamel.* | *Stage II*  *Enamel-dentin border reached.* | *Stage III*  *Outer third of the dentin.* | *Stage IV*  *Middle third of the dentin.* |
| II | Case II  A 15-year-old patient without orthodontic appliances. The patient has high caries activity and inadequate oral hygiene, has been attending the dental practice irregularly for routine oral examinations, and claims to brush once a day with a fluoridated toothpaste. | | | | |
| a | Monitoring |  |  |  |  |
| b | Oral hygiene instruction |  |  |  |  |
| c | Professional fluoride application |  |  |  |  |
| d | Restoration |  |  |  |  |
| e | Other, namely…  *(please fill in)* |  |  |  |  |
| *^a)^ The figures were reused from: Mejàre I, Sundberg H, Espelid I, Tveit B. Caries assessment and restorative treatment thresholds reported by Swedish dentists. Acta Odontol Scand. 1999; 57: 149–154. Mejàre et al. (1999).* | | | | | |

| 4a | The figures below show different stages of caries progression in the distal surface of tooth 46 in a 15-year-old patient. Would you please indicate your estimate of the average number of months that an approximal lesion needs to progress as outlined in the situations below? | | |
| --- | --- | --- | --- |
|  | 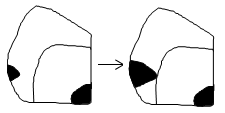 ^a)^ | 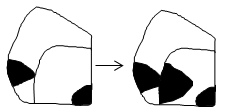 ^a)^ | |
|  | *Lesion progression in enamel from the outer layer of the enamel to the enamel-dentin border.* | *Lesion progression in dentin from the enamel-dentin border to the inner third part of the dentin.* | |
| I | Case I  A 15-year-old patient without orthodontic appliances. The patient has low caries activity and good oral hygiene, has been attending the dental practice regularly for routine oral examinations, and claims to brush twice a day with a fluoridated toothpaste. | | |
| a | 3 to 6 months | f | 3 to 6 months |
| b | 6 to 12 months | g | 6 to 12 months |
| c | 12 to 24 months | h | 12 to 24 months |
| d | 24 to 48 months | i | 24 to 48 months |
| e | > 48 months | j | > 48 months |
| *^a)^ The figures were reused from: Mejàre I, Sundberg H, Espelid I, Tveit B. Caries assessment and restorative treatment thresholds reported by Swedish dentists. Acta Odontol Scand. 1999; 57: 149–154. Mejàre et al. (1999).* | | | |

| 4b | The figures below show different stages of caries progression in the distal surface of tooth 46 in a 15-year-old patient. Would you please indicate your estimate of the average number of months that an approximal lesion needs to progress as outlined in the situations below? | | |
| --- | --- | --- | --- |
|  | 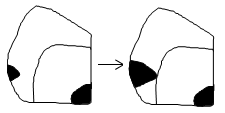 ^a)^ | 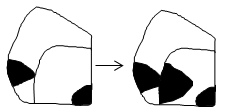 ^a)^ | |
|  | *Lesion progression in enamel from the outer layer of the enamel to the enamel-dentin border.* | *Lesion progression in dentin from the enamel-dentin border to the inner third part of the dentin.* | |
| II | Case II  A 15-year-old patient without orthodontic appliances. The patient has high caries activity and inadequate oral hygiene, has been attending the dental practice irregularly for routine oral examinations, and claims to brush once a day with a fluoridated toothpaste. | | |
| a | 3 to 6 months | f | 3 to 6 months |
| b | 6 to 12 months | g | 6 to 12 months |
| c | 12 to 24 months | h | 12 to 24 months |
| d | 24 to 48 months | i | 24 to 48 months |
| e | > 48 months | j | > 48 months |
| *^a)^ The figures were reused from: Mejàre I, Sundberg H, Espelid I, Tveit B. Caries assessment and restorative treatment thresholds reported by Swedish dentists. Acta Odontol Scand. 1999; 57: 149–154. Mejàre et al. (1999).* | | | |

| 5 | The figures below show three different stages of caries progression in the distal surface of tooth 46 as visible on a bitewing radiograph. How likely do you think caries lesions are to be cavitated in the following situations? | | | | |
| --- | --- | --- | --- | --- | --- |
|  | 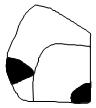^a)^ | ^a)^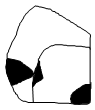 | | ^a)^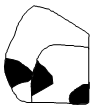 | |
|  | *Enamel-dentin border reached.* | *Outer third of the dentin.* | | *Middle third of the dentin.* | |
| a | 0-25% | f | 0-25% | k | 0-25% |
| b | 25-50% | g | 25-50% | l | 25-50% |
| c | 50-75% | h | 50-75% | m | 50-75% |
| d | 75-100% | i | 75-100% | n | 75-100% |
| e | 100% | j | 100% | o | 100% |
| *^a)^ The figures were reused from: Mejàre I, Sundberg H, Espelid I, Tveit B. Caries assessment and restorative treatment thresholds reported by Swedish dentists. Acta Odontol Scand. 1999; 57: 149–154. Mejàre et al. (1999).* | | | | | |

| 6 | How do you estimate the relationship between the actual depth of a caries lesion and the image on a radiograph? |
| --- | --- |
| a | The image on a radiograph underestimates the actual depth |
| b | The image on a radiograph equals the actual depth |
| c | The image on a radiograph overestimates the actual depth |

**Professional practice**

*Below are some questions about your professional practice as a dentist. They are, among other things, about the time you spend on various professional activities, your affinity with the treatment of children and your opinions on the treatment of young patients.*

| 7 | Below are some statements about the dental treatment of the youngest children (up to 6 years of age). Would you please indicate to what extent you agree or disagree with each statement? | | | | | |
| --- | --- | --- | --- | --- | --- | --- |
|  |  | I totally disagree | I mainly disagree | I do not agree nor disagree | I mainly agree | I totally agree |
| a | I get satisfaction from treating children. |  |  |  |  |  |
| b | I find it difficult to treat children. |  |  |  |  |  |
| c | The fees for the treatment of young children are inadequate |  |  |  |  |  |

| 8a | Do you get young patients referred from a fellow general dental practitioner within and/or outside your dental practice? If so, how many patients is that on average per month?  *You can disregard referrals in the context of orthodontics.* | |
| --- | --- | --- |
| a | Yes, on average … patients per month *(please fill in the number)* | |
| b | No | *Continue to question 9* |

| 8b | For which type of treatment(s) are young patients referred to you?  *Multiple options are possible.* |
| --- | --- |
| a | Total oral healthcare for the child |
| b | Curative treatments |
| c | Behavioral management techniques |
| d | Treatment under sedation or general anesthesia |
| e | Other, namely …. |

| 9a | Do you refer young patients to another oral healthcare provider, such as a fellow general dental practitioner within and/or outside your dental practice, an institution for oral healthcare for children (JTV ^a)^), a center for special dentistry (CBT ^b)^), or a pediatric dentist?  If so, how many patients is that on average per month?  *You can disregard referrals in the context of orthodontics.* | |
| --- | --- | --- |
| a | Yes, on average … patients per month *(please fill in the number)* | |
| b | No | *Continue to question 10* |
| *^a)^ ‘Instelling voor Jeugdtandverzorging’*  *^b)^ ‘Centrum voor Bijzondere Tandheelkunde’* | | |

| 9b | For which treatment(s) do you refer young patients?  *Multiple options are possible.* |
| --- | --- |
| a | Total oral healthcare for the child |
| b | Curative treatments |
| c | Behavioral management techniques |
| d | Treatment under sedation or general anesthesia |
| f | Other, namely …. |

| 10a | There is advice and a number of clinical guidelines available for oral healthcare for young patients, such as:   - *‘Richtlijn periodiek mondonderzoek’* (UMC St. Radboud, 2007) - *‘Advies cariëspreventie’* (Ivoren Kruis, 2011) - *‘Advies preventie fissuurcariës’* (Ivoren Kruis, 2012) - *‘Richtlijn mondzorg voor jeugdigen’* (KNMT/NVvK, 2012) - *‘Richtlijn mondzorg voor jeugdigen diagnostiek’* (KIMO, 2019) - *‘Richtlijn tandheelkundige radiologie’* (KNMT, 2013) - *‘Stappenplan Gewoon Gaaf’* (Ivoren Kruis, 2016)   Do you use one or more of these guidelines when treating young patients? |
| --- | --- |
| a | Never *Continue to question 11* |
| b | Sometimes |
| c | Regularly |
| d | Often |

| 10b | In which field do you (sometimes) use one or more of these guidelines?  *Multiple options are possible.* |
| --- | --- |
| a | Diagnostics |
| b | Radiographic diagnostics |
| c | Oral hygiene education and instruction |
| d | Sealants |
| e | Fluoride |
| f | Treatment of caries |
| g | Other, namely …. |

| 11 | Approximately how many hours do you spend on average per month on the following professional activities?  *Please fill in the number of hours.* | |
| --- | --- | --- |
| a | Post graduate courses | … hours per month |
| b | Peer consultation (study group, *‘IQual’*-group) | … hours per month |
| c | Reading professional literature (journals, books, e-learning) | … hours per month |

| 12 | Are you registered with the quality register dentists (KRT)? |
| --- | --- |
| a | Yes |
| b | No |

| 13 | Below are some methods that dentists can use to diagnose caries lesions in young patients.  Would you please indicate how frequently you use each of these methods? | | | | |
| --- | --- | --- | --- | --- | --- |
|  |  | never | sometimes | regularly | often/always |
| a | Bitewing radiographs |  |  |  |  |
| b | Transmitted light like ‘fiber optic transillumination’ FOTI |  |  |  |  |
| c | Removing plaque from the teeth |  |  |  |  |
| d | Use of dental loupes |  |  |  |  |
| e | Use of a probe |  |  |  |  |
| f | Quantitative detection methods like ‘digital imaging fiber optic transillumination’ (DIFOTI), ‘quantitative light-induced fluorescence’ (QLF), DIAGNOdent or ‘electrical conductance measurements’ (ECM) |  |  |  |  |
| g | Multi-function syringe |  |  |  |  |
| h | Visual inspection |  |  |  |  |
| i | Other, namely …. |  |  |  |  |

| 14 | Do you register the caries risk of a patient as regular part of a routine oral examination? |
| --- | --- |
| a | Yes |
| b | No |

| 15a | Four types of dentists ^a)^ are described below (Dentist A to D). Would you please read each of these descriptions carefully and indicate for each type to what extent you currently recognize yourself in the dentist described? You do this by placing an X at the corresponding percentage. |
| --- | --- |
|  | *EXAMPLE: You think that the described type of dentist does not resemble you very much, at most a little. You could say about 25%. You then put an X between 20 and 30. Like this:*  *X*  *0 10 20 30 40 50 60 70 80 90 100* |
|  | **Dentist A** is primarily aimed at limiting the damage caused by caries and replacing lost tissue, both in the primary and permanent dentition. If in doubt whether a caries lesion is cavitated, he will intervene restoratively and accept the risk of some unnecessary restorations. Prevention is important, but if it turns out that a patient and/or parents are not open to prevention, he does not see it as a dentist's task to motivate the patient towards prevention every time. |
| a | ***0 10 20 30 40 50 60 70 80 90 100*** |
|  | **Dentist B** is primarily aimed at preventing caries. He thinks it is a dentist’s first task to teach young patients and their parents the importance of prevention and to instruct them how self-care can be taken care of effectively. Restorative interventions are limited to prevent unnecessary restoration of healthy material. He accepts the risk of not treating some cavitated lesions. In case of doubt, he takes preventive measures, the effect of which is evaluated at the next routine oral examination. In the primary dentition, he tenaciously strives to stabilize a caries lesion through targeted preventive measures and/or proper advice to parents. |
| b | ***0 10 20 30 40 50 60 70 80 90 100*** |
|  | **Dentist C** thinks that an optimal treatment plan from a dental technical point of view should be drawn up for each patient. He only proposes treatments that he himself thinks are best, with the best possible dental prognosis, in which he believes 100%. He prefers not to treat patients who are not open to this. |
| c | ***0 10 20 30 40 50 60 70 80 90 100*** |
|  | **Dentist D** thinks that the wishes and possibilities of a patient are of great importance when drawing up a treatment plan. He informs the patient about the most desirable treatment from a dental point of view, but the patient is free to choose an alternative treatment option. Due to the input of a patient, different treatment plans can be drawn up for various patients with a similar oral situation. |
| d | ***0 10 20 30 40 50 60 70 80 90 100*** |
| *^a)^ The descriptions were based on:*   - *de Vries HH, Mellenbergh GJ, den Dekker J. Measurement of dentists’ treatment conceptions.. Ned Tijdschr Tandheelkd 1989; 96: 125-128.* - *den Dekker J. Behandelingsplanning in de tandartspraktijk. Academic thesis. Amsterdam: Universiteit van Amsterdam. 1990.* | |

| 15b | Which of the types of dentists described in question 15a do you most identify with? |
| --- | --- |
| a | Type A |
| b | Type B |
| c | Type C |
| d | Type D |

| 16 | At what age do you generally start the first routine oral examination of a child? |
| --- | --- |
| a | < 1 year old |
| b | Between 1 and 2 years of age |
| c | Between 2 and 3 years of age |
| d | Between 3 and 4 years of age |
| e | ≥ 4 years old |

| 17 | Do you generally apply a certain age limit before treating a cavity restoratively? |
| --- | --- |
| a | No |
| b | No, this depends on the cooperation of a child |
| c | Yes, principally I do not intervene restoratively in children younger than …years old  *(please fill in the age)* |

**Work and dental practice situation**

*Below are a number of questions about your work and dental practice situation. This includes your working week, the number of patients, practice busyness and working in a team.*

*The questions are about the dental practice with which you participate in this study.*

| 18 | How do you work in the dental practice? |
| --- | --- |
| a | As owner of the dental practice |
| b | As self-employed dentist (turnover-based salary) |
| c | As employed dentist (contract employee) |
| d | Other, namely ….  *(please explain)* |

| 19 | How many patients visit your dental practice at least once a year? What proportion of them is estimated to concern young patients?  *Please enter the number and then the percentage.* |
| --- | --- |
| a | … patients per year |
| b | … % of them concerns young patients (0 up to 17 years old) |

| 20 | How many patients do you personally treat (including those who only need routine oral examinations) on average per week? What proportion of them is estimated to concern young patients?  *Please enter the number and then the percentage.* |
| --- | --- |
| a | I treat … patients per week |
| b | … % of them concerns young patients (0 up to 17 years old) |

| 21 | How would you describe your personal workload in general? |
| --- | --- |
| a | I am too busy and not able to fulfill the care demand |
| b | I am able to fulfill the care demand by working overtime |
| c | I am able to fulfill the care demand within regular working hours |
| d | I am not busy enough and could fulfill a larger care demand |

| 22 | Would you like to indicate the number of people currently working in your dental practice per function in the second column and the total number of hours per function per week in the third column? | | |
| --- | --- | --- | --- |
|  |  | Number of persons | Total number of hours per week |
| a | Dentists (general dental practitioners and differentiated dentists) |  |  |
| b | Dental hygienists |  |  |
| c | Prevention assistants and dental assistants |  |  |
| d | Dental prosthetists and dental technicians |  |  |
| e | Practice manager |  |  |
| f | Receptionists and administrative staff |  |  |
| g | Other employees, namely …  *(please explain)* |  |  |

| 23 | For each of the oral healthcare services mentioned below, would you like to indicate who carries out these services for your young patients?  *Multiple options are possible.* | | | |
| --- | --- | --- | --- | --- |
|  | | Dentist | Dental hygienist | Prevention assistant |
| a | Routine oral examination |  |  |  |
| b | Making bitewing radiographs |  |  |  |
| c | Caries diagnosis |  |  |  |
| d | Treatment of small cavities |  |  |  |
| e | Oral hygiene instruction |  |  |  |
| f | Tooth cleaning |  |  |  |
| g | Sealing |  |  |  |
| h | Fluoride treatment |  |  |  |

| 24 | Do you and the dental care providers with whom you work mainly treat your own patients or does it regularly happen that you and the dental care providers with whom you work treat each other's patients? |
| --- | --- |
| a | Not applicable |
| b | Mainly our own patients |
| c | Regularly each other’s patients |

| 25 | Have agreements been made in your dental practice about a practice policy on the provision of care to young patients? |
| --- | --- |
| a | Yes |
| b | No |
| c | I don’t know |
| d | Not applicable |

**General characteristics**

*Below you will be asked some questions about your background.*

| 26 | What is your gender? |
| --- | --- |
| a | Male |
| b | Female |

| 27 | What is your year of birth? |
| --- | --- |
| a | <yyyy> |

| 28 | In what year did you graduate as a dentist? |
| --- | --- |
| a | <yyyy> |

| 29 | Where did you graduate as a dentist? |
| --- | --- |
| a | Amsterdam, ACTA |
| b | Amsterdam, Universiteit van Amsterdam |
| c | Amsterdam, Vrije Universiteit |
| d | Groningen |
| e | Nijmegen |
| f | Utrecht |
| g | Other, namely … |

**Finally**

*We would like to know whether there are regional differences in the care provided to young patients. The region is determined on the basis of a 2-digit postal code.*

| 30 | What are the first two numbers of your dental practice's postal code? |
| --- | --- |
| a | <..> |
