# Supplementary material for: The relationship between self-reported preventive and curative orientations of dentists and oral healthcare services provided to Dutch young patients: An observational study
Source: PLoS One. 2024 Jul 5;19(7):e0306403. doi: 10.1371/journal.pone.0306403 (PMC11226104; doi:10.1371/journal.pone.0306403)
Supplement: S1 Table — (DOCX) [file pone.0306403.s002.docx]

**S2 Table. General personal and professional characteristics participating general dental practitioners (GDPs).**

| Gender GDP  male | **n (%)**  25 (67.6) |
| --- | --- |
| Year of birth GDP | **Range / median**  1951-1989 / 1962 |
| Year of graduation | **Range / median**  1980-2013 / 1989 |
| Place of graduation  Amsterdam  Groningen  Nijmegen  Utrecht  abroad | **n (%)**  13 (35.1)  8 (21.6)  10 (27.0)  5 (13.5)  1 (2.7) |
| Owner of the dental practice  yes | **n (%)**  34 (91.9) |
| Number of patients per week | **Range / mean (sd)**  25-200 / 96.9 (41.9) |
| Percentage of patients <18 years old  range / mean (sd) | 10-95 / 21.4 (13.6) |
| Gets children referred for care or (specialized) treatment  yes | **n (%)**  3 (8.1) |
| Refers children for care or (specialized) treatment  yes | **n (%)**  21 (56.8) |
| The GDP  is able to fulfill the care demand by working overtime  is able to fulfill the care demand within regular working hours  is not busy enough and could fulfill a larger care demand | **n (%)**  5 (13.5)  30 (81.1)  2 (5.4) |
