# Supplementary material for: The relationship between self-reported preventive and curative orientations of dentists and oral healthcare services provided to Dutch young patients: An observational study
Source: PLoS One. 2024 Jul 5;19(7):e0306403. doi: 10.1371/journal.pone.0306403 (PMC11226104; doi:10.1371/journal.pone.0306403)
Supplement: S2 Table — (DOCX) [file pone.0306403.s003.docx]

**S3 Table. Opinions of general dental practitioners (GDPs) on the management of dental caries in the primary dentition.**

| Opinions of the participating GDPs on the management of different situations concerning dental caries in the primary dentition in a 6-year-old patient with one cavity. The child is quiet and cooperative.  *GDPs were asked to indicate what their caries management approach would be in each stage. Multiple answers were possible.* | | | | |
| --- | --- | --- | --- | --- |
| *^a)^* | 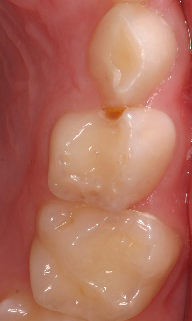 | 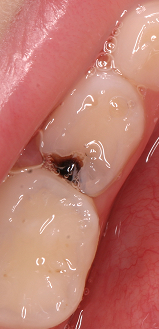 | 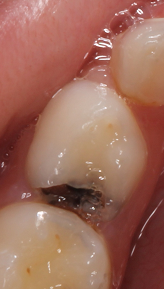 | 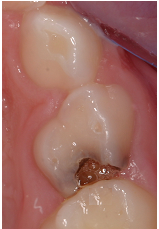 |
| *^b)^* | *Stage 1*  *A mesial occlusal cavity affecting less than half of the marginal ridge in the upper first primary molar tooth. The tooth is vital, and the child has no history of pain* | *Stage 2*  *A disto occlusal cavity affecting more than half of the marginal ridge in the lower first primary molar tooth. The tooth is vital, and the child has no history of pain.* | *Stage 3*  *A large distal occlusal cavity in the lower first primary molar tooth affecting more than half of the marginal ridge. The tooth is non-vital, but the child has no history of pain.* | *Stage 4*  *A large distal occlusal cavity in the upper first primary molar where more than half of the marginal ridge has been lost. The child is experiencing pain and has a swollen cheek.* |
|  | **Number of GDPs (%)** | | | |
| Monitoring | 14 (37.8) | 3 (8.1) | 8 (21.6) | 1 (2.7) |
| Prescribing/advising a painkiller | 0 (0.0) | 0 (0.0) | 0 (0.0) | 4 (10.8) |
| Prescribing antibiotics | 0 (0.0) | 0 (0.0) | 0 (0.0) | 0 (0.0) |
| Oral hygiene instruction | 31 (83.8) | 29 (78.4) | 29 (78.4) | 20 (54.1) |
| Professional fluoride application | 25 (67.6) | 19 (51.4) | 13 (35.1) | 7 (18.9) |
| Number of preventive items  0  1  2 | 5 (13.5)  8 (21.6)  24 (64.9) | 7 (18.9)  12 (32.4)  18 (48.6) | 8 (21.6)  16 (43.2)  13 (35.1) | 17 (45.9)  13 (35.1)  7 (18.9) |
| Non-restorative caries treatment | 18 (48.6) | 11 (29.7) | 5 (13.5) | 0 (0.0) |
| ART-restoration | 1 (2.7) | 4 (10.8) | 4 (10.8) | 0 (0.0) |
| Restoration | 13 (35.1) | 28 (75.7) | 13 (35.1) | 2 (5.4) |
| Prefab crown/ Hall technique | 1 (2.7) | 2 (5.4) | 3 (8.1) | 2 (5.4) |
| Opening the pulp chamber and allowing the tooth to drain | 0 (0.0) | 0 (0.0) | 3 (8.1) | 5 (13.5) |
| Pulpotomy | 0 (0.0) | 0 (0.0) | 7 (18.9) | 2 (5.4) |
| Tooth extraction | 0 (0.0) | 0 (0.0) | 8 (21.6) | 29 (78.4) |
| Refer for tooth extraction | 0 (0.0) | 0 (0.0) | 1 (2.7) | 4 (10.8) |
| Curative intervention | 31 (83.8) | 37 (100) | 30 (81.1) | 36 (97.3) |
| *^a)^ The photographs of the caries lesions were provided by the department of pediatric dentistry of ACTA.*  *^b)^ The descriptions were reused with minor adjustments from* *Tickle M, Threlfall AG, Pilkington L, Milsom KM, Duggal MS, Blinkhorn AS. Approaches taken to the treatment of young children with carious primary teeth: a national cross-sectional survey of general dental practitioners and paediatric specialists in England. Br Dent J. 2007 Jul28; 203(2): E4; discussion 102-103.* | | | | |
