# Supplementary material for: The relationship between self-reported preventive and curative orientations of dentists and oral healthcare services provided to Dutch young patients: An observational study
Source: PLoS One. 2024 Jul 5;19(7):e0306403. doi: 10.1371/journal.pone.0306403 (PMC11226104; doi:10.1371/journal.pone.0306403)
Supplement: S3 Table — (DOCX) [file pone.0306403.s004.docx]

**S4 Table. Opinions of general dental practitioners (GDPs) on the management of dental caries in an occlusal surface of a permanent tooth.**

| Opinions of the participating GDPs on the management of different stages of dental caries in an occlusal surface of a permanent tooth in a 15-year-old patient without orthodontic braces.  *GDPs were asked to indicate what their caries management approach would be in each stage. Multiple answers were possible.* | | | | |
| --- | --- | --- | --- | --- |
| *^a)^* 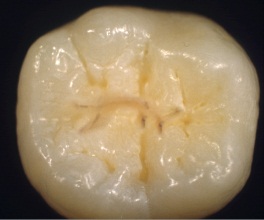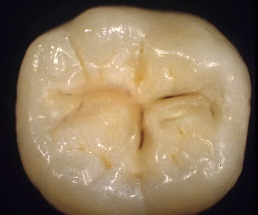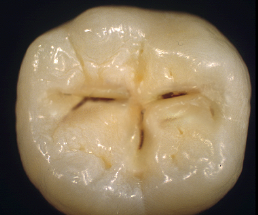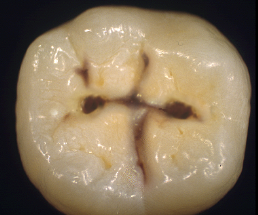 | | | | |
| *^b)^* | *Stage I*  *White / brownish discoloration in the enamel, no signs of cavitation. No radiographic signs of caries.* | *Stage II*  *Minor loss of tooth substance, with caries in the enamel. No radiographic signs of caries.* | *Stage III*  *Moderate loss of tooth substance. Caries in the outer third of the dentin according to the radiograph.* | *Stage IV*  *Considerable loss of tooth substance. Caries up to the middle third of the dentin according to the radiograph.* |
| **A. Low caries risk ^1)^** | **Number of GDPs (%)** | | | |
| Monitoring | 31 (83.8) | 22 (59.5) | 4 (10.8) | 1 (2.7) |
| Oral hygiene instruction | 25 (67.6) | 25 (67.6) | 24 (64.9) | 22 (59.5) |
| Professional fluoride application | 17 (45.9) | 18 (48.6) | 12 (32.4) | 12 (32.4) |
| Sealant | 14 (37.8) | 23 (62.2) | 10 (27.0) | 2 (5.4) |
| Number of preventive items  0  1  2  3 | 6 (16.2)  11 (29.7)  15 (40.5)  5 (13.5) | 5 (13.5)  8 (21.6)  14 (37.8)  10 (27.0) | 9 (24.3)  13 (35.1)  12 (32.4)  3 (8.1) | 12 (32.4)  15 (40.5)  9 (24.3)  1 (2.7) |
| Restoration | 0 (0.0) | 1 (2.7) | 29 (78.4) | 35 (94.6) |
| **B. High caries risk ^2)^** | **Number of GDPs (%)** | | | |
| Monitoring | 24 (64.9) | 13 (35.1) | 1 (2.7) | 1 (2.7) |
| Oral hygiene instruction | 36 (97.3) | 36 (97.3) | 36 (97.3) | 36 (97.3) |
| Professional fluoride application | 28 (75.7) | 28 (75.7) | 22 (59.5) | 22 (59.5) |
| Sealant | 18 (48.6) | 19 (51.4) | 3 (8.1) | 2 (5.4) |
| Number of preventive items  0  1  2  3 | 1 (2.7)  5 (13.5)  16 (43.2)  15 (40.5) | 3 (8.1)  22 (59.5)  12 (32.4) | 1 (2.7)  13 (35.1)  21 (56.8)  2 (5.4) | 1 (2.7)  13 (35.1)  22 (59.5)  1 (2.7) |
| Restoration | 0 (0.0) | 7 (18.9) | 36 (97.3) | 36 (97.3) |
| *^a)^ The photographs were reused from: Espelid I, Tveit AB, Mejàre I, Sundberg H, Hallonsten AL. Restorative treatment decisions on occlusal caries in Scandinavia. Acta Odontol Scand. 2001; 59: 21–27.*  *^b)^ The descriptions were reused from: Mejàre I, Sundberg H, Espelid I, Tveit B. Caries assessment and restorative treatment thresholds reported by Swedish dentists. Acta Odontol Scand. 1999; 57: 149–154. Mejàre et al. (1999).*  *^1)^ The patient has low caries activity and has been attending the dental practice for a routine oral examination on a regular basis, has good oral hygiene and claims to brush twice a day with a fluoridated toothpaste.*  *^2)^ The patient has high caries activity and inadequate oral hygiene, has been attending the dental practice for a routine oral examination on an irregular basis and claims to brush once a day with a fluoridated toothpaste.* | | | | |
