# Supplementary material for: The relationship between self-reported preventive and curative orientations of dentists and oral healthcare services provided to Dutch young patients: An observational study
Source: PLoS One. 2024 Jul 5;19(7):e0306403. doi: 10.1371/journal.pone.0306403 (PMC11226104; doi:10.1371/journal.pone.0306403)
Supplement: S4 Table — (DOCX) [file pone.0306403.s005.docx]

**S5 Table. Opinions of general dental practitioners (GDPs) on the management of dental caries in an approximal surface of a permanent tooth.**

| Opinions of the participating GDPs on the management of different stages of dental caries in an approximal surface of a permanent tooth in a 15-year-old patient without orthodontic braces.  *GDPs were asked to indicate what their caries management approach would be in each stage. Multiple answers were possible.* | | | | |
| --- | --- | --- | --- | --- |
| 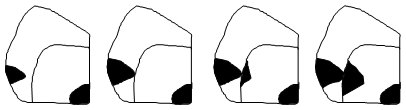  *^a)^* | | | | |
|  | *Stage I*  *Inner half of the enamel.* | *Stage II*  *Enamel-dentin border reached.* | *Stage III*  *Outer third of the dentin.* | *Stage IV*  *Middle third of the dentin.* |
| **A. Low caries risk ^1)^** | **Number of GDPs (%)** | | | |
| Monitoring | 0 (0.0) | 34 (91.9) | 16 (43.2) | 1 (2.7) |
| Oral hygiene instruction | 34 (91.9) | 35 (94.6) | 33 (89.2) | 33 (89.2) |
| Professional fluoride application | 22 (59.5) | 25 (67.6) | 25 (67.6) | 20 (54.1) |
| Number of preventive items  0  1  2 | 2 (5.4)  14 (37.8)  21 (56.8) | 2 (5.4)  10 (27.0)  25 (67.6) | 4 (10.8)  8 (21.6)  25 (67.6) | 4 (10.8)  13 (35.1)  20 (54.1) |
| Restoration | 0 (0.0) | 4 (10.8) | 21 (56.8) | 37 (100) |
| **B. High caries risk ^2)^** | **Number of GDPs (%)** | | | |
| Monitoring | 35 (94.6) | 27 (73.0) | 4 (10.8) | 3 (8.1) |
| Oral hygiene instruction | 37 (100.0) | 36 (97.3) | 34 (91.9) | 34 (91.9) |
| Professional fluoride application | 29 (78.4) | 29 (78.4) | 23 (62.2) | 22 (59.5) |
| Number of preventive items  0  1  2 | 8 (21.6)  29 (78.4) | 9 (24.3)  28 (75.7) | 1 (2.7)  15 (40.5)  21 (56.8) | 2 (5.4)  14 (37.8)  21 (56.8) |
| Restoration | 2 (5.4) | 9 (24.3) | 36 (97.3) | 37 (100) |
| *^a)^ The figures were reused from: Mejàre I, Sundberg H, Espelid I, Tveit B. Caries assessment and restorative treatment thresholds reported by Swedish dentists. Acta Odontol Scand. 1999; 57: 149–154. Mejàre et al. (1999).*  *^1)^ The patient has low caries activity and has been attending the dental practice for a routine oral examination on a regular basis, has good oral hygiene and claims to brush twice a day with a fluoridated toothpaste.*  *^2)^ The patient has high caries activity and inadequate oral hygiene, has been attending the dental practice for a routine oral examination on an irregular basis and claims to brush once a day with a fluoridated toothpaste.* | | | | |
