# Supplementary material for: The relationship between self-reported preventive and curative orientations of dentists and oral healthcare services provided to Dutch young patients: An observational study
Source: PLoS One. 2024 Jul 5;19(7):e0306403. doi: 10.1371/journal.pone.0306403 (PMC11226104; doi:10.1371/journal.pone.0306403)
Supplement: S5 Table — A. Lesion progression in an approximal surface of a permanent tooth in a 15-year-old patient without orthodontic braces. Estimated time needed for progression from the first into the second stage. B. Estimated percentage of cavitated lesions in different stages of the caries process. C. Estimation of the relationship between the actual depth of a caries lesion and the image on a bitewing radiograph. (DOCX) [file pone.0306403.s006.docx]

**S6 Table. Estimated severity of caries lesions by general dental practitioners (GDPs).**

| **A. Lesion progression in an approximal surface of a permanent tooth in a 15-year-old patient without orthodontic appliances. Estimated time needed for progression from the first into the second stage.** | | | | | | | | | | |
| --- | --- | --- | --- | --- | --- | --- | --- | --- | --- | --- |
| 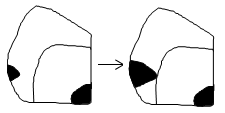^a)^ | | | | | | 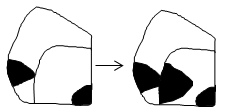 | | | | |
| *Lesion progression in enamel from the outer layer to the enamel-dentin border.* | | | | | | *Lesion progression in dentin from the enamel-dentin border to the inner third part of the dentin.* | | | | |
|  | | **Number of GDPs (%)** | | | |  | | **Number of GDPs (%)** | | |
|  |  | **Low caries risk ^1)^** | | **High caries risk ^2)^** | |  |  | **Low caries risk ^1)^** | | **High caries risk ^2)^** |
| 3 to 6 months | | 0 (0.0) | | 8 (21.6) | | 3 to 6 months | | 0 (0.0) | | 9 (24.3) |
| 6 to 12 months | | 3 (8.1) | | 20 (54.1) | | 6 to 12 months | | 8 (21.6) | | 20 (54.1) |
| 12 to 24 months | | 15 (40.5) | | 8 (21.6) | | 12 to 24 months | | 18 (48.6) | | 8 (21.6) |
| 24 to 48 months | | 11 (29.7) | | 1 (2.7) | | 24 to 48 months | | 9 (24.3) | | 0 (0.0) |
| > 48 months | | 8 (21.6) | | 0 (0.0) | | > 48 months | | 2 (5.4) | | 0 (0.0) |
| *^1)^ The patient has low caries activity and has been attending the dental practice for a routine oral examination on a regular basis, has good oral hygiene and claims to brush twice a day with a fluoridated toothpaste.*  *^2)^ The patient has high caries activity and inadequate oral hygiene, has been attending the dental practice for a routine oral examination on an irregular basis and claims to brush once a day with a fluoridated toothpaste.* | | | | | | | | | | |
| **B. Estimated percentage of cavitated lesions in different stages of the caries process.** | | | | | | | | | | |
| 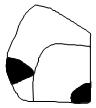  ^a)^ | | | 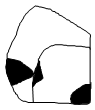 | | | | 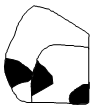 | | | |
| *Enamel-dentin border reached.* | | | *Outer third of the dentin.* | | | | *Middle third of the dentin.* | | | |
|  | **Number of GDPs (%)** | |  | | **Number of GDPs (%)** | |  | | **Number of GDPs (%)** | |
| 0-25% | 29 (78.4) | | 0-25% | | 9 (24.3) | | 0-25% | | 0 (0.0) | |
| 25-50% | 5 (13.5) | | 25-50% | | 12 (32.4) | | 25-50% | | 4 (10.8) | |
| 50-75% | 3 (8.1) | | 50-75% | | 10 (27.0) | | 50-75% | | 8 (21.6) | |
| 75-100% | 0 (0.0) | | 75-100% | | 6 (16.2) | | 75-100% | | 16 (43.2) | |
| 100% | 0 (0.0) | | 100% | | 0 (0.0) | | 100% | | 9 (24.3) | |
| **C. Estimation of the relationship between the actual depth of a caries lesion and the image on a bitewing radiograph.** | | | | | | | | | | |
| **Statement** | | | | | | | **Number of GDPs (%)** | | | |
| The image on a radiograph underestimates the actual depth | | | | | | | 29 (78.4) | | | |
| The image on a radiograph equals the actual depth | | | | | | | 5 (13.5) | | | |
| The image on a radiograph overestimates the actual depth | | | | | | | 3 (8.1) | | | |
| *^a)^ The figures were reused from: Mejàre I, Sundberg H, Espelid I, Tveit B. Caries assessment and restorative treatment thresholds reported by Swedish dentists. Acta Odontol Scand. 1999; 57: 149–154. Mejàre et al. (1999).* | | | | | | | | | | |
