# Supplementary material for: The relationship between self-reported preventive and curative orientations of dentists and oral healthcare services provided to Dutch young patients: An observational study
Source: PLoS One. 2024 Jul 5;19(7):e0306403. doi: 10.1371/journal.pone.0306403 (PMC11226104; doi:10.1371/journal.pone.0306403)
Supplement: S6 Table — (DOCX) [file pone.0306403.s007.docx]

**S7 Table. Professional opinions and behaviors of the participating general dental practitioners (GDPs).**

| GDPs recognizing themselves in type, mean percentage recognition ^1)^  A – restorative  B – preventive  C – directive  D – communicative | **% (sd)**  16.9 (15.9)  65.1 (25.9)  18.8 (19.4)  73.5 (23.3) |
| --- | --- |
| GDPs recognizing themselves mostly in type ^1)^  A – restorative  B – preventive  C – directive  D – communicative | **N (%)**  0 (0.0)  12 (32.4)  2 (5.4)  23 (62.2) |
| Treatment of young children derives satisfaction  I totally agree  I mainly agree  I do not agree nor disagree  I mainly disagree  I totally disagree | **N (%)**  15 (40.5)  19 (51.4)  2 (5.4)  1 (2.7)  0 (0.0) |
| Treatment of young children is difficult  I totally agree  I mainly agree  I do not agree nor disagree  I mainly disagree  I totally disagree | **N (%)**  0 (0.0)  6 (16.2)  6 (16.2)  20 (54.1)  5 (13.5) |
| The fees for the treatment of young children are inadequate  I totally agree  I mainly agree  I do not agree nor disagree  I mainly disagree  I totally disagree | **N (%)**  2 (5.4)  1 (2.7)  15 (40.5)  16 (43.2)  3 (8.1) |
| Use of clinical guidelines and recommendations  often  regularly  sometimes  never  Used clinical guidelines and recommendations concerning  diagnostics  radiographic diagnostics  oral hygiene education and instruction  sealants  fluoride  treatment of dental caries  otherwise, namely trauma | **N (%)**  15 (40.5)  9 (24.3)  7 (18.9)  6 (16.2)  **N (%)**  18 (48.6)  20 (54.1)  29 (78.4)  16 (43.2)  22 (59.5)  24 (64.9)  2 (5.4) |
| Use of (additional) diagnostic methods  bitewing radiographs  transmitted light  plaque removal  dental loupes  probe  quantitative detection methods  multi-function syringe  visual inspection | **N (%)**  36 (97.3)  13 (35.1)  36 (97.3)  29 (78.4)  34 (91.9)  1 (2.7)  37 (100)  37 (100) |
| Registration of caries risk as regular part of a routine oral examination  yes | **N (%)**  20 (54.1) |

| First routine oral examination  ≤ 1 year old  between 1 and 2 years old  between 2 and 3 years old  between 3 and 4 years old | **N (%)**  2 (5.4)  16 (43.2)  16 (43.2)  3 (8.1) |
| --- | --- |
| Age limit for a restorative intervention of a carious lesion  no  no, this depends on the cooperation of a child  yes, principally I do not intervene restoratively in children younger than 3 (n=1) or 4 years old (n=2) | **N (%)**  5 (13.5)  29 (78.4)  3 (8.1) |
| Number of hours per month spent on professional activities  post graduate courses  peer consultation  reading professional literature  total | **range / mean (sd)**  1-24 / 5.0 (4.5)  0-16 / 3.5 (3.4)  1-20 / 5.3 (3.8)  2-40 / 13.8 (9.0) |
| Registered with the quality register dentists (KRT)  yes | **N (%)**  30 (81.1) |
| *Descriptions based on de Vries et al. (1989) and den Dekker (1990).*  ***GDP A*** *is primarily aimed at limiting the damage caused by caries and replacing lost tissue, both in the primary and permanent dentition. If he is doubting whether a caries lesion is cavitated, he will intervene restoratively and accept the risk of some unnecessary restorations. Prevention is important, but if it turns out that a patient and/or parents are not open to prevention, he does not see it as a GDP's task to motivate the patient towards prevention every time.*  ***GDP B*** *is primarily aimed at preventing caries. He thinks it is a GDP's first task to teach young patients and their parents the importance of prevention and to instruct them how self-care can be taken care of effectively. Restorative interventions are limited to prevent unnecessary restoration of healthy material. He will accept the risk of not treating some cavitated lesions. In case of doubt, he takes preventive measures, the effect of which is evaluated at the next routine oral examination. In the primary dentition, he tenaciously strives to stabilize a caries lesion through targeted preventive measures and/or proper advice to parents.*  ***GDP C*** *thinks that an optimal treatment plan from a dental technical point of view should be drawn up for each patient. He only proposes treatments that he himself thinks are best, with the best possible dental prognosis, in which he believes 100%. He prefers not to treat patients who are not open to this.*  ***GDP D*** *thinks that the wishes and possibilities of a patient are of great importance when drawing up a treatment plan. He informs the patient about the most desirable treatment from a dental point of view, but the patient is free to choose an alternative treatment option.* *Due to the input of a patient, different treatment plans can be drawn up for various patients with a similar oral situation.* | |
