# Supplementary material for: The relationship between self-reported preventive and curative orientations of dentists and oral healthcare services provided to Dutch young patients: An observational study
Source: PLoS One. 2024 Jul 5;19(7):e0306403. doi: 10.1371/journal.pone.0306403 (PMC11226104; doi:10.1371/journal.pone.0306403)
Supplement: S7 Table — (DOCX) [file pone.0306403.s008.docx]

**S8 Table. Characteristics of the participating dental practices.**

| Number of patients in dental practice | **range / mean (sd)**  710-9000 / 3054 (1882) | | | |
| --- | --- | --- | --- | --- |
| Percentage of patients <18 years in dental practice | **range / mean (sd)**  10-35 / 19.8 (5.9) | | | |
| Employees, ≥ 1, mean number of employees, mean number of hours per week  GDPs  oral hygienists (OH)  prevention assistants (PA) and dental assistants | **≥ 1**  37 (100)  21 (56.8)  35 (94.6) | **mean (sd)**  2.3 (1.8)  1.2 (1.8)  3.4 (2.2) | **hrs per wk(sd)**  60.1 (38.1)  27.3 (32.7)  88.3 (66.9) | |
| Executors of procedure  routine oral health examination  bitewing radiographs  caries diagnostics  treatment of small cavities  oral hygiene instruction  professional tooth cleaning  professional fluoride application  sealants | **GDP N (%)**  37 (100)  33 (89.2)  37 (100)  37 (100)  32 (86.5)  31 (83.8)  23 (62.2)  30 (81.1) | **OH N (%)**  5 (13.5)  5 (13.5)  7 (18.9)  4 (10.8)  16 (43.2)  16 (43.2)  12 (32.4)  11 (29.7) | | **PA N (%)**  1 (2.7)  17 (45.9)  1 (2.7  0 (0.0)  26 (70.3)  22 (59.5)  27 (73.0)  15 (40.5) |
| GDPs treating their own patients  only own patients  mainly own patients  regularly each other’s patients | **N (%)**  13 (35.1)  12 (32.4)  12 (32.4) | | | |
| Practice policy on the provision of care to young patients  yes  no  not applicable | **N (%)**  22 (59.5)  9 (24.3)  6 (16.2) | | | |
| Mean number of inhabitants per GDP in region | **range / mean (sd)**  1035-2994 / 2140 (454) | | | |
