# Supplementary material for: The relationship between self-reported preventive and curative orientations of dentists and oral healthcare services provided to Dutch young patients: An observational study
Source: PLoS One. 2024 Jul 5;19(7):e0306403. doi: 10.1371/journal.pone.0306403 (PMC11226104; doi:10.1371/journal.pone.0306403)
Supplement: S8 Table — (DOCX) [file pone.0306403.s009.docx]

**S9 Table. Variables predicting preventive and curative care patterns (based on p-value <0.05).**

| *Predictor* | *occasional preventive care ^1)^* | *regular preventive care ^1)^* | *curative treat-ment(s) in 1 year ^2)^* | *curative treatments in several years ^2)^* |
| --- | --- | --- | --- | --- |
| **Self-reported preventive and curative orientation GDP** |  |  |  |  |
| - Preventive orientation |  |  | n/a | n/a |
| - Curative orientation | n/a | n/a | ✓ | ✓ |
| **Patient characteristics** |  |  |  |  |
| - Age category Jan 2013 | ✓ | ✓ | ✓ | ✓ |
| - Gender patient |  |  |  |  |
| - Income category neighborhood | ✓ | ✓ | ✓ | ✓ |
| - Number of years included in study | ✓ | ✓ | ✓ | ✓ |
| **Personal characteristics GDP** |  |  |  |  |
| - Gender GDP |  |  |  |  |
| - Year of graduation |  |  |  |  |
| - Place of graduation | ✓ | ✓ |  |  |
| **Caries-related opinions GDP** |  |  |  |  |
| - Estimated lesion progression rate | n/a | n/a | ✓ | ✓ |
| - Estimated percentage cavitated lesions | n/a | n/a |  |  |
| - Depth of radiographic lesion is an underestimate | n/a | n/a | ✓ | ✓ |
| **Professional beliefs and behaviors GDP** |  |  |  |  |
| - Practice style |  |  | ✓ | ✓ |
| - Experiences an obstacle in the treatment of young children | ✓ | ✓ | n/a | n/a |
| - Use of clinical guidelines | ✓ |  | ✓ | ✓ |
| - Use of (additional) diagnostic methods |  |  | ✓ |  |
| - Registration of caries risk as regular part of a ROE |  |  | ✓ | ✓ |
| - Age first ROE | n/a | n/a |  |  |
| - Age limit for a restorative intervention |  |  | n/a | n/a |
| **Professional characteristics GDP** |  |  |  |  |
| - Owner of the dental practice |  |  |  |  |
| - Number of patients per week GDP |  |  | n/a | n/a |
| - Percentage of young patients (<18 yrs) GDP | ✓ |  |  |  |
| - Refers children for (specialized) care |  |  |  |  |
| - Personal workload | ✓ | ✓ | ✓ | ✓ |
| - Professional activities |  | ✓ |  |  |
| **Characteristics dental practice** |  |  |  |  |
| - Number of patients dental practice | ✓ | ✓ | ✓ | ✓ |
| - Percentage of young patients (<18 yrs) dental practice | ✓ | ✓ | n/a | n/a |
| - Number of tasks executed by a dental hygienist and/or prevention assistant |  |  | n/a | n/a |
| - Practice policy on the provision of care to young patients | ✓ | ✓ | ✓ | ✓ |
| - Mean number of inhabitants per GDP in region | ✓ | ✓ |  |  |
| ^1)^ Reference category was no preventive care  ^2)^ Reference category was no curative treatments | | | | |
| n/a = not applicable | | | | |
